# Thrombus-targeted nano-agents for NIR-II diagnostic fluorescence imaging-guided flap thromboembolism multi-model therapy

**DOI:** 10.1186/s12951-022-01649-6

**Published:** 2022-10-14

**Authors:** Zichen Cao, Xinyu Zhang, Zheng Wei, Chuanhui Song, Huihui Zou, Jianchuan Ran, Hongbo Zhang, Diya Xie, Shengwei Han, Yufeng Wang, Yu Cai, Wei Han

**Affiliations:** 1 Department of Oral Medicine, Nanjing Stomatological Hospital, Medical School of Nanjing University, 30 Zhongyang Road, Nanjing, 210008 China; 2 Department of Oral and Maxillofacial Surgery, Nanjing Stomatological Hospital, Medical School of Nanjing University, No 30 Zhongyang Road, Nanjing, 210008 China; 3 Pediatric Dentistry, Nanjing Stomatology hospital, Medical School of Nanjing University, No 30 Zhongyang road, Nanjing, 210008 China; 4 Institute of Translational Medicine, The Affiliated Drum Tower Hospital of Nanjing University Medical School, Nanjing, 210008 China; 5 Center for Rehabilitation Medicine, Rehabilitation & Sports Medicine Research Institute of Zhejiang Province, Department of Rehabilitation Medicine, Zhejiang Provincial People’s Hospital (Affiliated People’s Hospital, Hangzhou Medical College), Hangzhou, 310014 Zhejiang China

**Keywords:** Oral and maxillofacial surgery, Flap repair, Thrombus-targeted, Phototherapy, NIR-II

## Abstract

In oral and maxillofacial surgery, flap repair is essential to the quality of postoperative life. Still, thrombosis is fatal for the survival of the flaps. Besides, some postoperative thrombotic diseases, such as pulmonary embolism, also intimidate patients’ life. The traditional diagnostic methods are still limited by a large amount of hardware and suffer from inconvenience, delay, and subjectivity. Moreover, the treatments mainly rely upon thrombolytics, such as urokinase (UK) plasminogen activator, which may cause bleeding risk, especially intracerebral hemorrhage. Herein, a kind of poly (lactic-co-glycolic acid) (PLGA) nanoparticles (NPs) containing a first near-infrared window (NIR-I) phototheranostic agent Y8 and urokinase plasminogen activator (UK) as the core, and modified with the fibrin-targeting peptide Gly–Pro–Arg–Pro–Pro (GPRPP) were developed for the flap and postoperative thromboembolism treatment (named GPRPP-Y8U@P). The conjugated molecule Y8 endows GPRPP-Y8U@P with the capacity of NIR-II imaging and excellent photothermal/photodynamic therapeutic effects. In vivo experiments demonstrated that GPRPP-Y8U@P could quickly locate thrombus by NIR-II fluorescence imaging, and semi-quantitative analysis of the embolized blood vessels' paraffin section verified its thrombolytic efficiency. Additionally, the urokinase trapped in the NPs would not result in nonspecific bleeding, tremendously improving physical security and curative effects with minimizing side effects. Overall, the advantages of GPRPP-Y8U@P, such as precise localization of the thrombus, thrombus ablation in the site, and mild side effects, demonstrated the attractiveness of this approach for effective clinical monitoring of thrombus therapy.

**Supplementary Information:**

The online version contains supplementary material available at 10.1186/s12951-022-01649-6.

## Introduction

A thrombus is a blood clot forming in a vessel, blocking the vessel and leading to life-threatening events such as ischemic stroke, arteriovenous thrombosis, deep vein thrombosis (DVT), and pulmonary thromboembolism (PTE). Thrombus has caused the majority of morbidity and mortality worldwide [1–3]. In oral and maxillofacial surgery, the thrombus is responsible for most flap reconstruction failures [4–6] and some postoperative complications (for example, DVT and PTE) [7, 8]. Clinical monitoring is the principal diagnosis method for the flap thrombus but lacks sensitivity and may not timely find the flap abnormality. Surgical exploration is the primary rescue method but will bring secondary damage and psychological burden to the patients [9]. For postoperative thrombotic diseases, the diagnosis gold standard (ultrasound for lower extremity or computed tomography angiography) needs a specialist. Besides, it may result in the embolus falling off and worsen the patient's symptoms owning to moving the patient back and forth. The treatment can be divided into surgical thrombectomy and medicine thrombolysis [9]. Surgical thrombectomy is usually expensive and with a high recurrence rate but low long-term efficacy. Medicine thrombolysis may cause bleeding due to the nonspecific activating fibrinolytic system, poor targeting ability, and persistent administration because of its short half-life [10, 11]. Therefore, it is prominent to propose a workable treatment to promptly detect thrombosis and effective thrombolysis in thrombus events, including flap reconstruction and postoperative thrombus complications.

For thrombus diagnosis, molecular fluorescence imaging has shown excellent vitality in thrombus detection, with higher sensitivity and earlier diagnosis, raising the exciting application prospect of overcoming the limitation of large-scale imaging instruments [12]. Significantly, many targeted molecular imaging probes have been proposed via common modification of targeted peptides [12–19]. Besides, Tinglu confirmed that indocyanine green (ICG) angiography could predict flap thrombosis bedside [13], indicating that fluorescence molecular imaging is more suitable for thrombus detection in emergent clinical situations. Even the imaging did not show satisfactory results. Noteworthily, the recently developed second near-infrared window (NIR-II, 900–1700 nm) fluorescence imaging has a much higher tissue penetration depth and signal-to-noise ratio (SNR) than the first near-infrared (NIR-I, 780–900 nm) imaging. Additionally, owing to minimized tissue autofluorescence, tissue scattering or absorption, and decreased interference by fluorescent proteins, NIR-II imaging provides many benefits, including maximum permissible exposure, high resolution, and quick feedback [14–17]. Hence, we believe NIR-II fluorescence imaging would exhibit more brilliant imaging capability and offer easy-read images to determine thrombosis [18, 19].

For thrombus therapy, based on the shortcomings covered above, researchers have been exploring various methods to reduce the risks of drug thrombolysis. For instance, different targeted delivery strategies have been studied. However, they did not alter the nature of drug thrombolysis, and the risk of drug leakage still exists. High-intensity ultrasound was also introduced to treat thrombus. However, ultrasound would break the thrombus instead of ablation for mild therapy [20–22]. Besides ultrasound, photothermal therapy (PTT) [23, 24] and photodynamic cure (PDT) [25] have been found to have an efficient therapeutic effect for thrombolytic treatment. Recent studies proved that PDT combined with PTT thrombolysis could avoid re-embolization with high spatiotemporal selectivity [25]. To our best knowledge, numerous NIR-II agents have been validated in biomedical applications with outstanding PDT and PTT properties [26–28]. With the imaging faculty and PDT/PTT effect, we believe eminent NIR-II phototheranostics of thrombus may thrive within the foreseeable future.

**Scheme 1:**
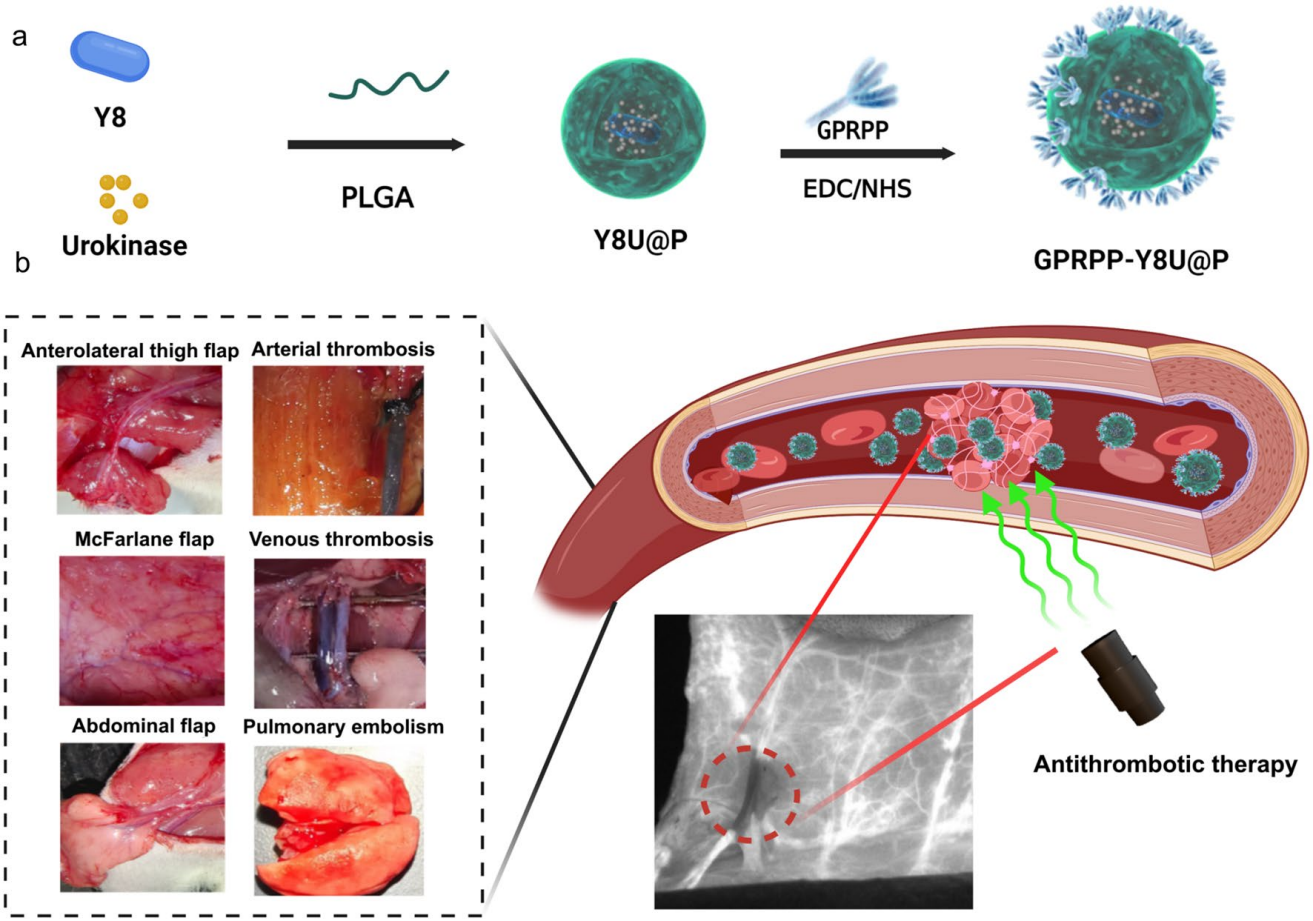
GPRPP-Y8U@P played as an integrated nanomedicine for thrombus therapy. **a** The Y8U@P was fabricated by the double emulsion method, and the targeting peptide GPRPP was connected via EDC/NHS to synthesize GPRPP-Y8U@P. **b** The GPRPP-Y8U@P had surpassing characteristics in antithrombotic therapy and was confirmed by the six animal models: (1) Three flap animal models: Anterolateral thigh flap, McFarlane flap, Abdominal flap. The flap thrombus was established by applying ferric chloride to the donor's vessel. (2) Three thrombus animals: arterial thrombosis, venous thrombosis, and pulmonary embolism. (3) GPRPP-Y8U@P could intuitively detect thrombus by fluorescence imaging, utilizing photothermal, photodynamic, and UK trip effects that allowed for a firmly thrombolytic effect. (4) The thrombolytic procedure minimized bleeding risk due to controlled by Laser

Inspired by the distinctive advantages of NIR-II imaging and the combined effect of thrombolysis, we sought to develop a comprehensive antithrombotic therapy strategy, including flap monitoring and immediate thrombolysis in situ. In this study, the rational design of a thrombus-targeted agent named GPRPP-Y8U@P was formulated by taking advantage of the surface modification of GPRPP and good biocompatibility of PLGA incorporating a NIR-II agent Y8 (Additional file 1: Fig. S1) and thrombolytics. After systemic injection, the nanoparticles targeted fibrin and detected thrombus formation with significant NIR-II fluorescence imaging. Its multimodal thrombolysis nature upon NIR laser irradiation indicated that the curative effect could be amplified and integrated to obtain rapid vascular recanalization. The ability to detect thrombus and multimodal thrombolysis ability of the GPRPP-Y8U@P were thoroughly verified in vitro and in various animal models (Scheme 1b). And hence, it had potential benefits for the future development of flap postoperative management. Moreover, this established antithrombotic therapeutic strategy heralded a new era in thrombosis research.

## Result and discussion

### Synthesis and characterizations of the NPs

**Fig. 1:**
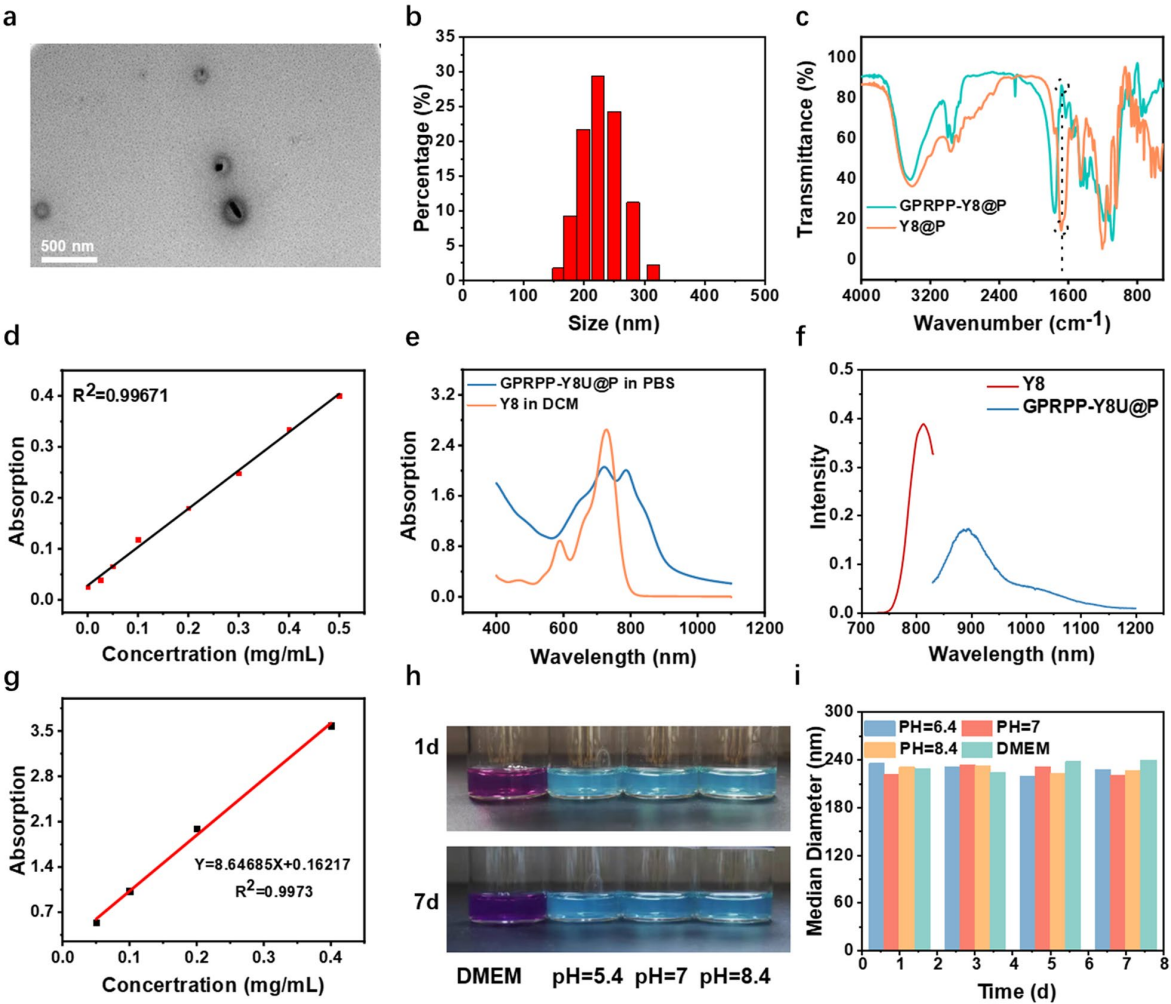
**a** The TEM image of GPRPP-Y8U@P. **b** The size distribution of GPRPP-Y8U@P. **c** The Fourier transform infrared spectroscopy (FTIR) spectra of GPRPP-Y8@P (upper) and Y8@P (low). Based on the curve of Y8@P, the characteristic peak of the GPRPP-Y8@P is about 1650.31 cm^−1^, which is caused by the stretching vibration of the amide bond, proving GPRPP was connected successfully. **d** The BCA standard curve of GPRPP in MES to calculate GPRPP loading rate. **e** The fluorescence spectroscopy of Y8 in DCM and GPRPP-Y8U@P in PBS. **f** The fluorescence emission spectroscopy of Y8 in DCM and GPRPP-Y8U@P in PBS. **g** The absorption of GPRPP-Y8U@P at different concentrations and the absorption coefficient was calculated at 8.64685. **h** Pictures of GPRPP-Y8U@P in corresponding storage media at room temperature. The storage media were DMEM and ddH_2_O at different pH values (pH = 5.4, 7, 8.4). **i** Stability of GPRPP-Y8U@P after seven days' preservation

The Y8U@P was prepared using a classic double emulsification solvent evaporation method. GPRPP peptides were coupled to the NPs using standard carbodiimide chemistry [29] to obtain GPRPP-Y8U@P (Scheme 1). The transmission electron microscope (TEM) and dynamic light scattering (DLS) proved that the NPs were about 220 nm in a circular shape (Fig. 1a, b and Additional file 1: Fig. S2a, b). As shown in Fig. 1c, GPRPP was successfully linked to the surface of the NPs confirmed by Fourier transform infrared spectroscopy (FTIR) with a 69.67% loading rate, according to the standard curve of GPRPP (Fig. 1d). The UK was successfully loaded into the NPs with a 58.35% loading rate through BCA Protein Assay Kit, while the Y8 was 81.8% by way of the standard curve of Y8 in dichloromethane (DCM) (Additional file 1: Fig. S2c, d). After forming NPs, the GPRPP-Y8U@P NPs with a J-aggregative state manifested a bathochromic shift in the absorption and emission spectrum [30], making it a NIR-II imaging agent. The ultraviolet–visible (UV–vis) spectrum absorption spectrum for Y8 was at 728 nm, while GPRPP-Y8U@P also showed a bathochromic shift to 788 nm (Fig. 1e). Furthermore, the fluorescence emission spectrum for Y8 was at 812 nm, and the peak was observed at about 900 nm for GPRPP-Y8U@P, demonstrating that GPRPP-Y8U@P could well bathochromic-shift for fluorescence imaging at NIR-II region (Fig. 1f). Moreover, the absorption coefficients in PBS were also calculated as 8.64 (Fig. 1g). The size and exterior of GPRPP-Y8U@P were monitored every other day for a week to examine the storage stability at DMEM and ddH_2_O (pH = 5.4, 7, 8.4) in a dry, cool place at room temperature, which showed superb long-term storage stability of GPRPP-Y8U@P (Fig. 1h, i).

Drug release was also studied. 5 mg Y8U@P in 5 mL PBS was exposed to 808 nm irradiation (0 or 0.6 w/cm^2^) for 2 h. The supernatant was measured by a BCA kit every 10 min. as shown in Additional file 1: Fig. S3, the drug was released slowly and got a total of about 60% cumulative release after 2 h with 0 w/cm^2^ irradiation. However, NIR could accelerate it. Under 0.6 w/cm^2^, it could release 50% within 30 min, and the maximum release amount was about 80%. The aperture of the PLGA shell led to a slow release without NIR [31]. These results proved that the nanoparticles could realize rapid release to facilitate thrombolysis through NIR exposure.

### Photothermal and photodynamic capacity of GPRPP-Y8U@P

**Fig. 2:**
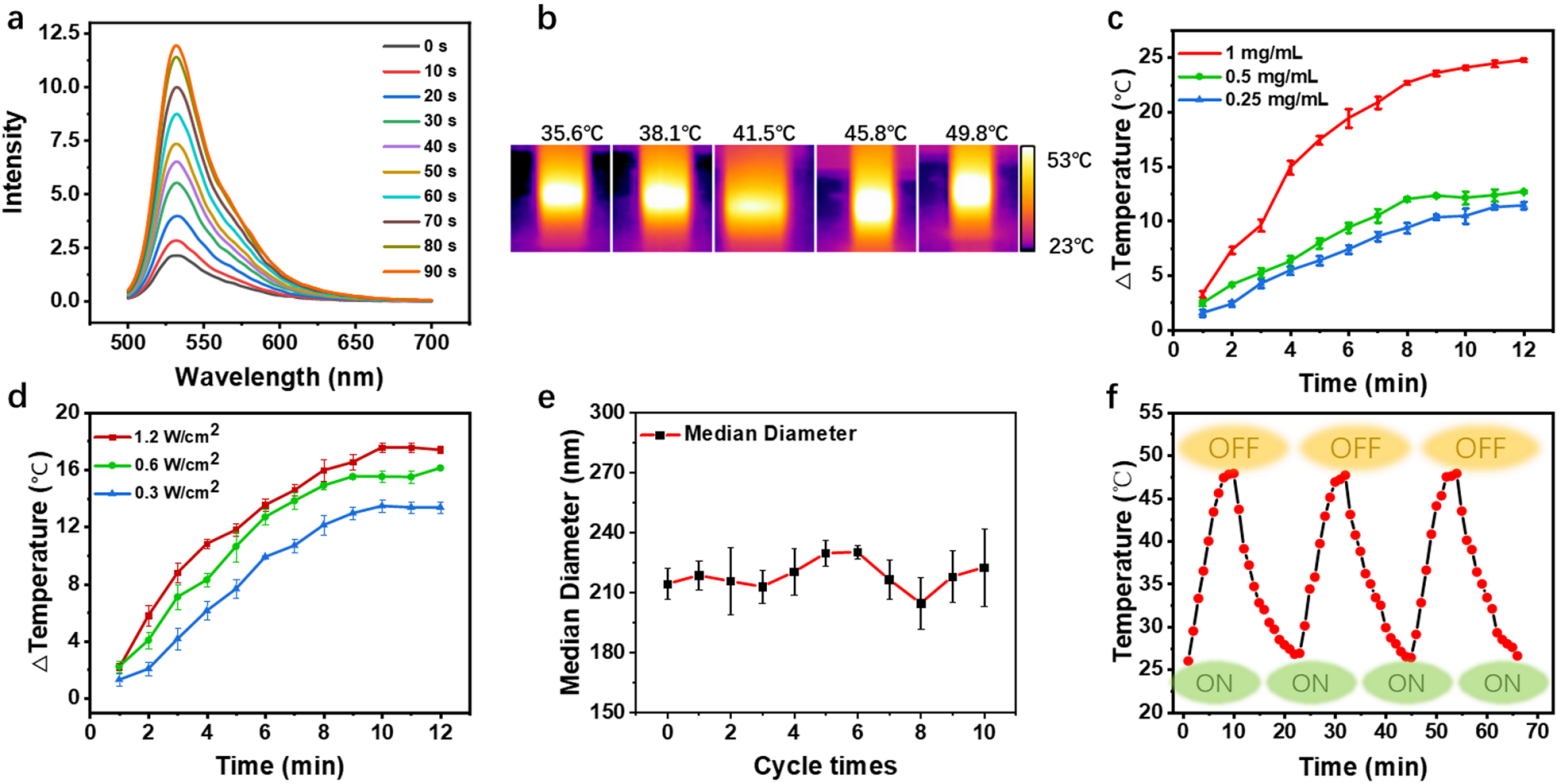
**a** Photodynamic Performance of GPRPP-Y8U@P (10 μg/mL GPRPP-Y8U@P + 5 μM SOSG) and irradiated (808 nm Laser, 1 W/cm^2^). SOSG was used to detect the singlet oxygen generation according to the protocol (excitation and emission wavelengths were 488 and 525 nm). **b** Pictures recorded the temperature change over time at the set concentration of 1 mg/mL. **c** The concentration-dependent PTT of GPRPP-Y8U@P. The temperature change with time under 0.6 w/cm^2^. **d** The power-dependent PTT of GPRPP-Y8U@P. The temperature change with time at the concentration of 1 mg/mL. **e** Particle size stability of GPRPP-Y8U@P after ten photothermal cycles. **f** Heating–cooling curve of GPRPP-Y8U@P

The photodynamic capacity of GPRPP-Y8U@P was explored as follows, the singlet oxygen production of GPRPP-Y8U@P under laser irradiation was detected by singlet oxygen sensor green (SOSG) at 525 nm emission wavelength. Moreover, the emission intensity significantly increased from 2.1 to 11.9 in just 90 s under laser irradiation (Fig. 2a), which means GPRPP-Y8U@P could be a highly PDT agent with a high singlet oxygen generation capacity. Singlet oxygen could inhibit coagulation and activate fibrinolysis by inactivating some clotting factors and the main fibrinolysis inhibitors [25, 32]. Therefore, the above data supported that GPRPP-Y8U@P could give full play to photodynamic antithrombotic therapy. To further investigate the photothermal effect of GPRPP-Y8U@P, after continuous irradiation, the GPRPP-Y8U@P displays terrific concentration-dependent (0.25–1 mg/mL) and power-dependent (0.3–1.2 W/cm^2^) photothermal effect, which could increase about 25 ℃ within 10 min (Fig. 2c, d). We also measured its photothermal conversion efficiency (about 34.58%) as the reported method, which was relatively high than most of the mentioned photothermal agents (< 30%) [33, 34].

Furthermore, the DLS results and the heating–cooling cycles curve did not exhibit significant differences after GPRPP-Y8U@P was irradiated to the peak temperature and cooled down at room temperature ten times (Fig. 2e, f). It denoted that GPRPP-Y8U@P has good photothermal stability. Due to photothermal-potentiated thrombus penetration, mediating disintegration of a fibrin clot, and precisely controlled release of UK in situ, the data above favor that GPRPP-Y8U@P was an advanced photodynamic and photothermal combined theranostic platform for PDT/PTT thrombolytic therapy [35–37].

### Imaging capability and tissue penetration depth in vitro

**Fig. 3:**
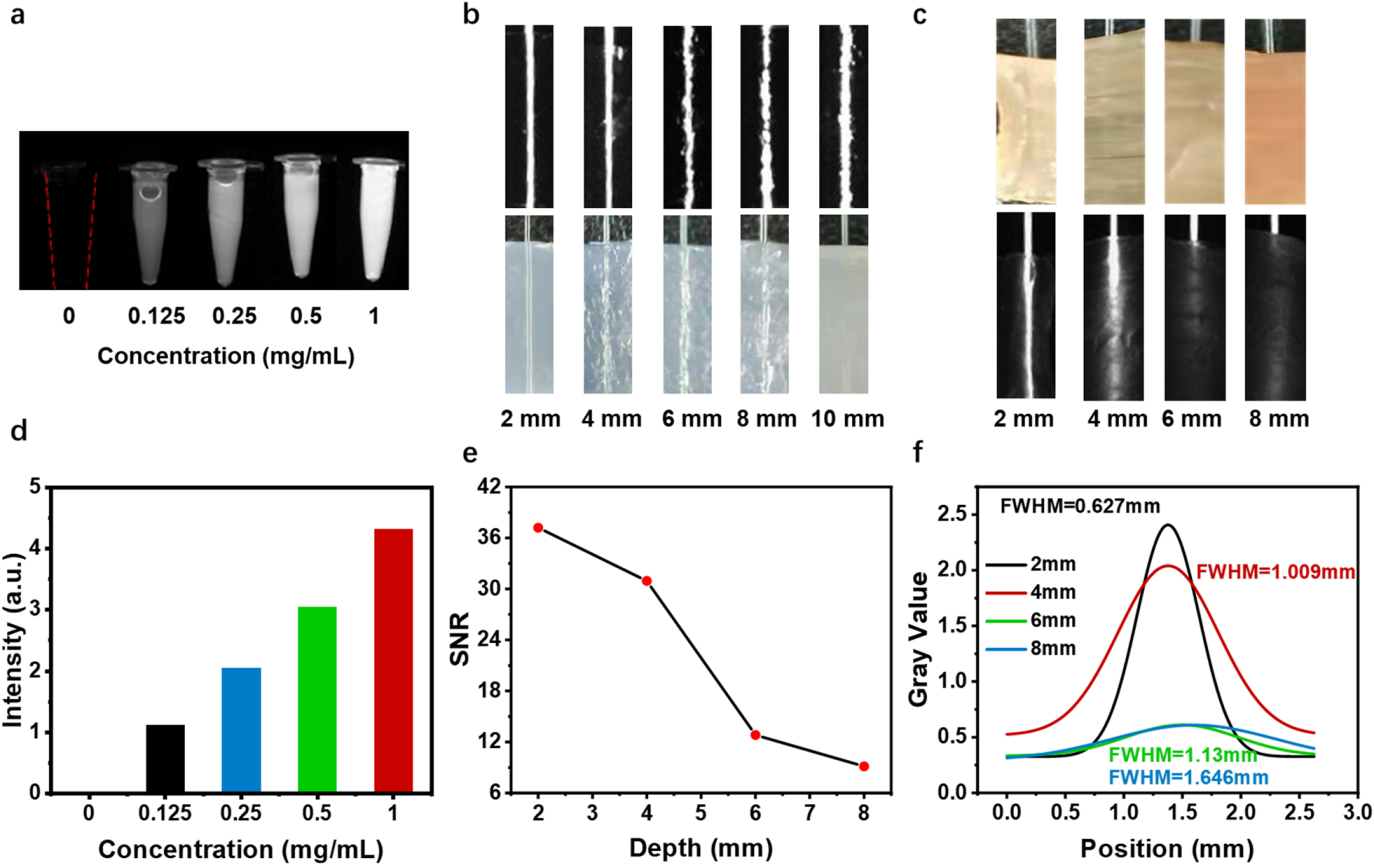
GPRPP-Y8U@P has outstanding NIR-II imaging ability and tissue penetration depth. **a** NIR-II imagines of GPRPP-Y8U@P under 1064 nm at different concentrations. **b** The penetration depth is under 3% agar. **c** The penetration depth under the chicken breast block. **d** Quantification data of fluorescence signal intensity in (**a**). **e** The Signal–Noise-Ratio under the corresponding chicken breast block in (**c**). **f** The full width at half maximum in (**c**)

NIR-II fluorescence imaging capacity was tested under an In Vivo Imaging System at different concentrations, which showed concentration-dependent imaging capacity. Figure 3a and d show that even at a low 0.125 mg/mL concentration, it still has excellent imaging intensity. As for tissue penetration depth, 0.1 mg/mL GPRPP-Y8U@P was put into capillary tubes with a 1 mm diameter. Then, they were first placed under the agar block, exhibiting excellent tissue penetration depth (Fig. 3b). Furthermore, they were also placed under the fresh chicken breast muscle (Fig. 3c), the SNR (Fig. 3e), and full width at half-maximum (FWHM) (Fig. 3f) of capillary tubes were calculated. Even at 6 mm of chicken breast muscle with a high signal-to-noise ratio, the maximum was 0.627 mm at 2 mm. These results concluded that GPRPP-Y8U@P offers better tissue penetration depth and signal-to-noise ratio than ICG, laying the foundation for application in vivo imaging.

### Targeting ability in vitro

**Fig. 4:**
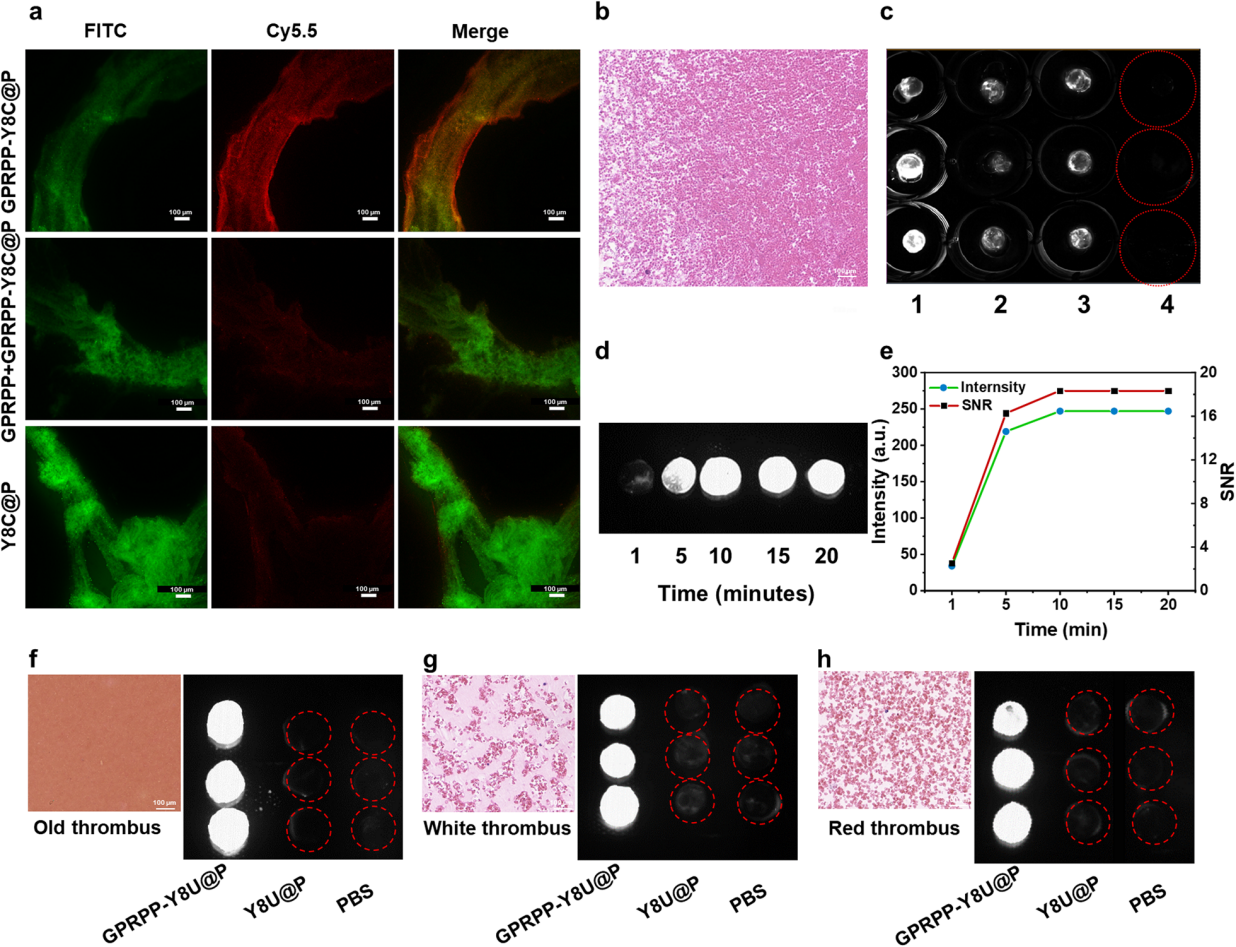
**a** The binding ability of NPs to fibrin-FITC in vitro under the confocal microscope; images of fibrin clots: FITC signal image of fibrin clots (left), Cy5.5 signal image of GPRPP-Y8C@P binding (middle), and images depicting clot binding (right). Scale bar = 100 μm. **b** HE staining images of fresh thrombus. Scale bar = 100 μm **c** The binding ability of GPRPP-Y8U@P to the fresh thrombus. Groups 1–4 represented GPRPP-Y8U@P, Y8U@P, GPRPP + GPRPP-YU@P, and PBS groups. **d** GPRPP-Y8U@P was incubated with thrombus under a static state at different times. **e** The intensity and SNR of every group were quantified. **f**–**h** The HE staining of the old, white and red thrombus and the binding ability assay of NPs to the thrombus

Three kinds of experimental models were designed to explore the binding ability of GPRPP-Y8U@P to the thrombus. First, we verified whether the GPRPP peptide could bind to fibrin. As shown in Fig. 4a, GPRPP-Y8C@P showed vigorous red fluorescence signal intensity with the green signal of fibrin under the confocal microscope. In contrast, the other two groups showed little red fluorescence signal, proving that GPRPP had a remarkable affinity for fibrin and is the base of the thrombus-targeted agent GPRPP-Y8U@P. The same results were observed in the fresh blood clots targeting assay (Fig. 4b, c): the blood clots dealt by GPRPP-Y8U@P possessed a strong fluorescence signal, while the other two groups with a little signal. The results were repeated in red, white, or old Thrombus (Fig. 4f–h). Thrombi can be classified as red and white, and they prefer formation at different sites [38]. We prepared different thrombi and verified that GPRPP-Y8U@P could also target them. Establishing an old thrombus proved that GPRPP-Y8U@P could detect thrombus for some chronic patients susceptible to a thrombus. This information confirmed that GPRPP-Y8U@P could target various kinds of blood clots with broad prospects in clinical application. Besides, in the static model, we designed different incubation times and found that only 5 min GPRPP-Y8U@P takes to realize thrombus imaging (Fig. 4d, e). The quick check for identification of thrombus was consistent with literature reports [39]. This result should own to the high affinity for fibrin of GPRPP. It can be explained by the equilibrium dissociation constant (K_d_): CREKA has a K_d_ of 6 μM, while GPRPP has a K_d_ of around 8 nM [40]. Thus, these experiments not only attested that GPRPP could bind fibrin but also proved it undoubtedly a thrombus-targeted agent with splendid targeting ability, implying it has the foundation of thrombolysis in situ and reduced the risk of bleeding.

### Thrombolysis activity in vitro

**Fig. 5:**
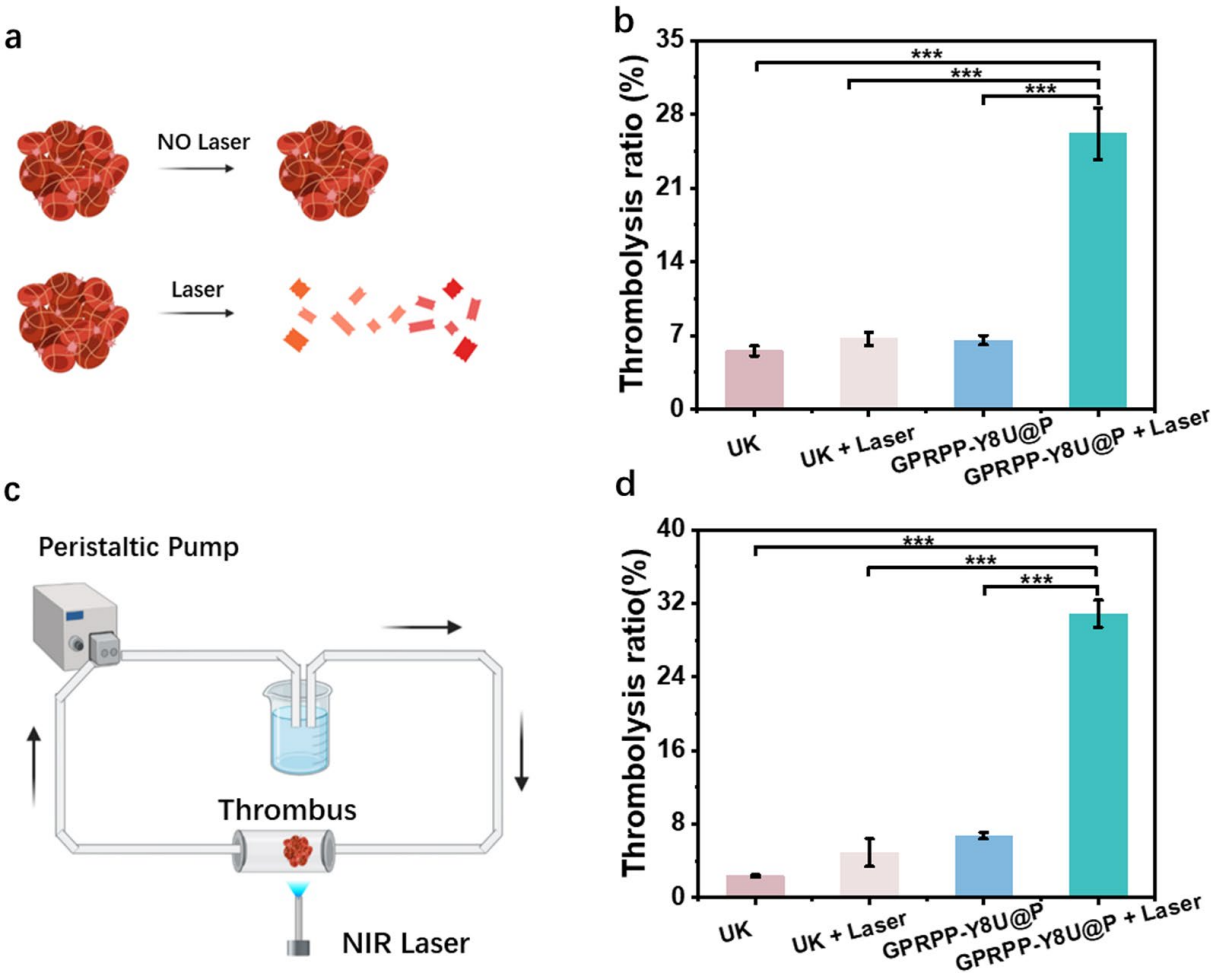
Thrombolysis evaluation in vitro. **a** Static thrombolysis model. **b** The thrombolysis ratio under different conditions in the static thrombolysis model. **c** A 3D vascular model was constructed to verify the dynamic thrombolysis effect. **d** The thrombolysis ratio under different conditions in the dynamic thrombolysis model. The above thrombus was incubated with 1 mg/mL GPRPP-Y8U@P or 0.58 mg/mL UK. Asterisks indicate significantly differences (*P < 0.01, **P < 0.001, ***P < 0.0001)

The thrombolytic capacity in vitro was appraised via weight loss ratio after treatment. The blood clots were placed in glass bottles for the static or dynamic thrombolysis and randomly divided into four groups (n = 3 per group): the UK group, the UK + Laser group, the GPRPP-Y8U@P group, and the GPRPP-Y8U@P + Laser group (Fig. 5). The concentration of GPRPP-Y8U@P was 1 mg/mL while the UK was 0.58 mg/mL since the content of the UK among each group was the same. The group without Laser was incubated for 30 min alone, and the group with Laser received 0.6 W/cm^2^ irradiation (808 nm) for 30 min. After treatment, the surface moisture of blood clots was carefully removed with filter paper. The blood clots were weighted again, marked as the final weight. Then the weight loss ratio data were obtained to assess thrombolytic capacity, and we could conclude from Fig. 5 that: (1) the thrombolytic capacity of GPRPP-Y8U@P was about 24% higher than in the UK. (2) without the Laser, the thrombolytic capacity of GPRPP-Y8U@P was small. We speculated that the Laser activates the thrombolysis procedure. (3) PDT/PTT-mediated thrombolysis is practicable. (4) the flowing velocities may explain why the thrombolysis rate was higher in the dynamic model than in the static model.

### Blood compatibility evaluation

**Fig. 6:**
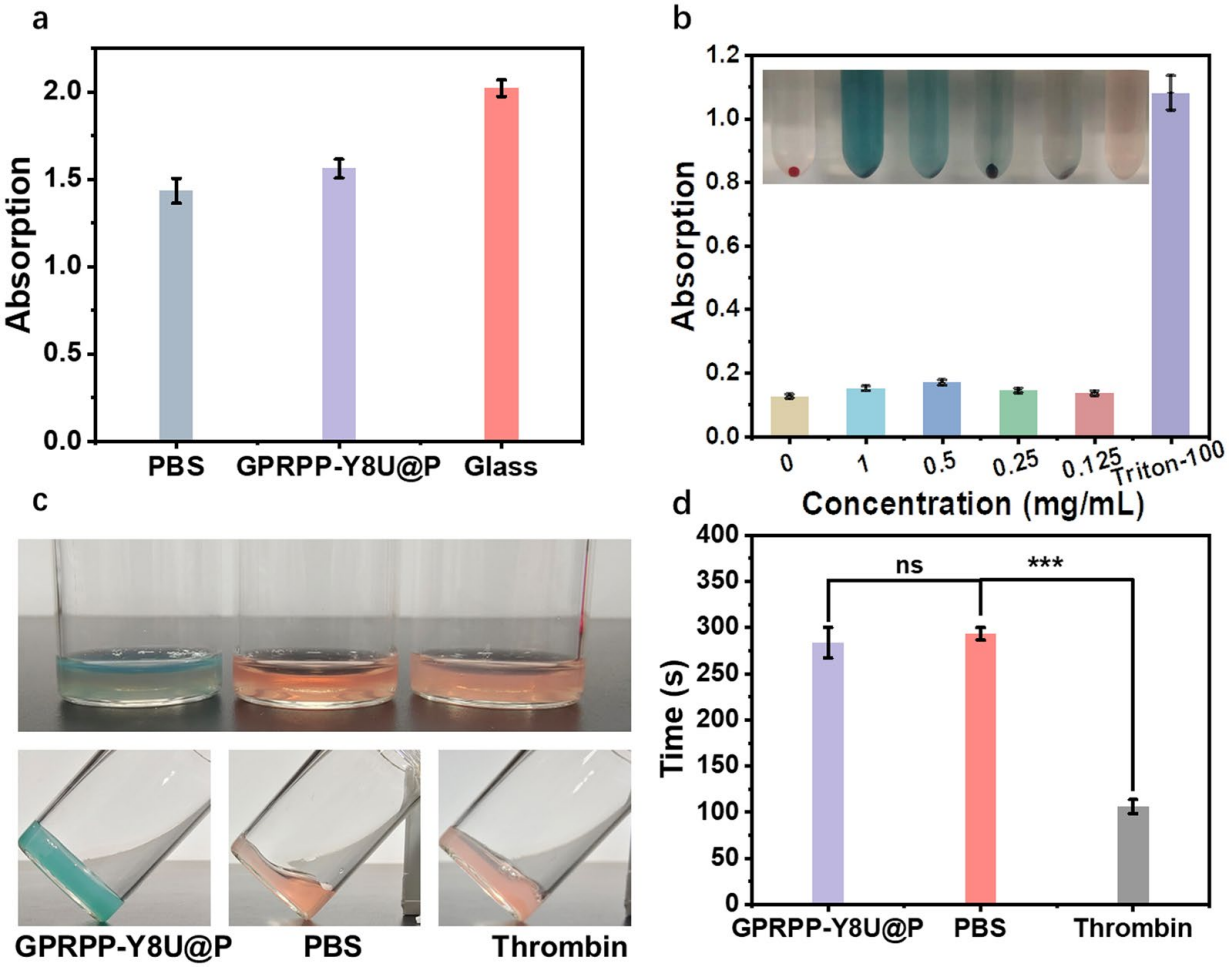
Blood compatibility evaluation: **a** platelet adhesion assay: platelet-rich plasma (PRP) incubated with nothing as the negative control. At the same time, the PRP incubated with the glass powder was a positive control. **b** Hemolysis assay: 2% red blood cell suspension was incubated with PBS, GPRPP-Y8U@P (1, 0.5, 0.25, 0.125 mg/mL), and 0.1% Triton-100. **c** Pictures of plasma recalcification time assay. Pictures of plasma with corresponding processing (upper). Pictures of plasma solidification (underside). **d** Blood clotting time: the time of sample solidification was recorded as PRT. Asterisks indicate significantly differences (*P < 0.01, **P < 0.001, ***P < 0.0001)

In this part, three tests were arranged to evaluate the blood compatibility of GPRPP-Y8U@P. First, platelet adhesion was a statistic used to measure harm to platelet by GPRPP-Y8U@P and quantified by LDH Cytotoxicity Assay Kit (Fig. 6a). The platelet-rich plasma was added to EP tubes containing nothing, glass powder, or GPRPP-Y8U@P. The negative control was the empty tube, while the glass group was positive. The amount of GPRPP-Y8U@P and glass both were 1 mg. No apparent damage caused by GPRPP-Y8U@P was observed when the suspension was transferred 96-well plate and measured by the LDH kit. Second, the hemolysis assay offered data to measure the damage caused by different concentrations of GPRPP-Y8U@P to red blood cells (RBCs) (Fig. 6b). Hemolysis was reported as the absorbance at 540 nm, with the hemolysis caused by PBS set at 0% and caused by 0.1% Triton-100 set at 100%. The data indicated that the GPRPP-Y8U@P has no apparent damage to RBCs, even at 1 mg/mL. Third, the time of fiber appearance for each group with different treatments following the addition of calcium chloride was determined as plasma recalcification time (PRT). PRT can be utilized to compute whether GPRPP-Y8U@P can activate the intrinsic coagulation pathway (Fig. 6c, d). Fortunately, minor damage was noticed again. Summarizing the results above, we hold the opinion that it is safe to use GPRPP-Y8U@P in blood and will not coagulant.

### Cytotoxicity test

**Fig. 7:**
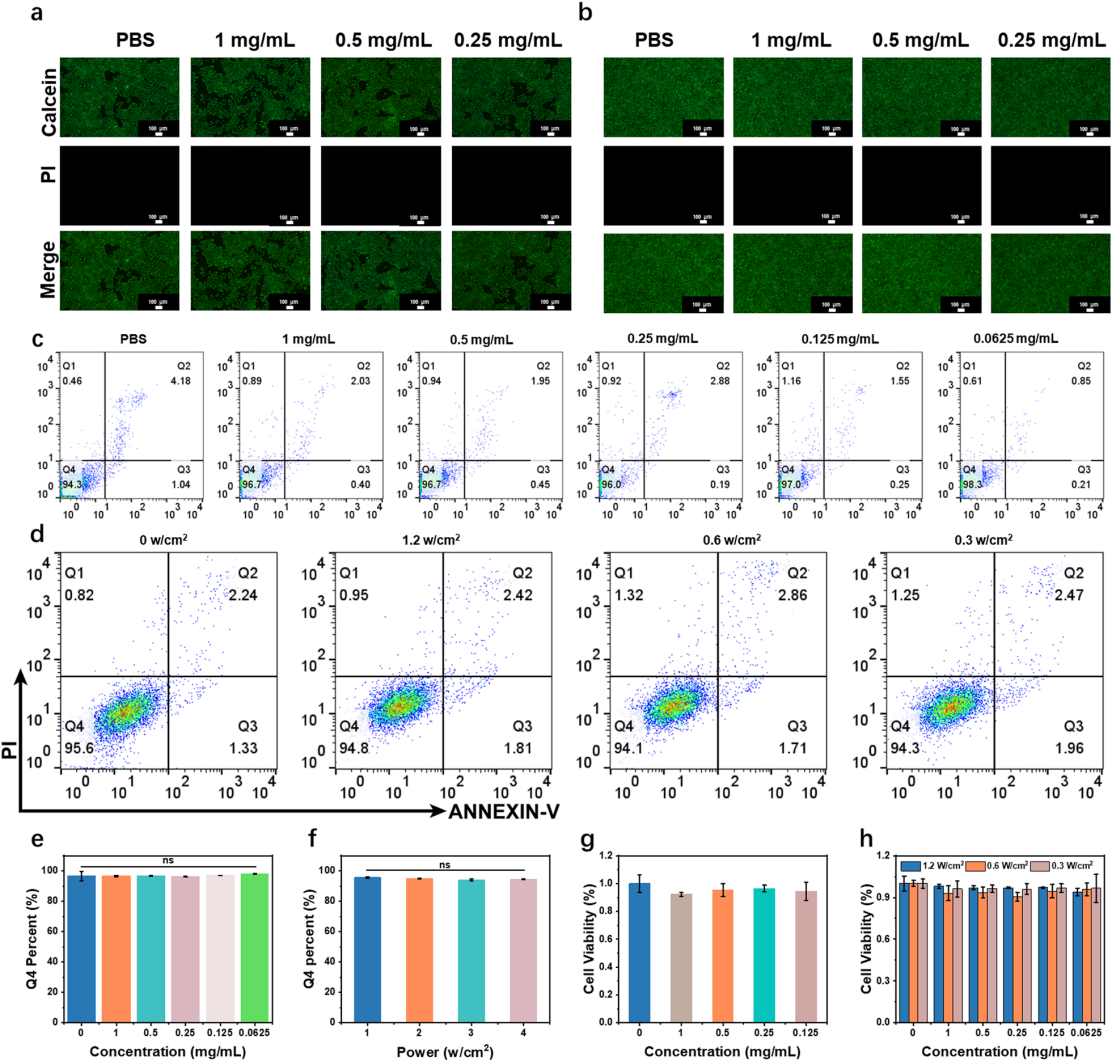
**a** HUVECs viability after simple co-incubation with GPRPP-Y8U@P by fluorescence microscopy. **b** HUVECs viability after laser co-incubation with GPRPP-Y8U@P by fluorescence microscopy. **c** Apoptosis of HUVEC under co-incubation with GPRPP-Y8U@P at different concentrations by Flow cytometer. **d** Apoptosis of HUVEC under laser co-incubation with GPRPP-Y8U@P at 1 mg/mL concentration under different powers. **e** Quantitative analysis of Q4 shown in (**c**) (mean ± SD, n = 3). **f** Quantitative analysis of Q4 shown in (**d**) (mean ± SD, n = 3). **g** HUVECs viability after simple co-incubation with GPRPP-Y8U@P by a microplate reader. **h** HUVECs viability after laser co-incubation with GPRPP-Y8U@P by a microplate reader

Next, we explored the cytotoxicity of GPRPP-Y8U@P through two kinds of cells via three methods (CCK-8 assay, Calcein-AM/PI Double Stain Kit, and Annexin V-FITC/PI apoptosis detection kit) under two conditions (simple co-incubation or laser co-incubation). First, live/dead staining (Calcein-AM/PI Double Stain Kit) (Fig. 7a, b) indicated that there was barely a red signal in these groups. These results were repeated by flow cytometry (FCM) (Fig. 7c, d). The quantitative analysis of living cells in Q4 also showed great cytocompatibility (Fig. 7e, f). Moreover, cell viability in experiment groups measured by CCK-8 was still > 90% (Fig. 7g, h). The data of the experiment group, simple co-incubation or laser co-incubation, was similar to the PBS group, so we concluded that GPRPP-Y8U@P barely led to cell death and inhibited cell proliferation here. Although under gentle laser irritation, the cell viability is similar to the PBS group.

The same results were observed in Raw264.7 (a mice monocyte/macrophage cell line) (Additional file 1: Fig. S4). To sum up, cytotoxicity caused by the GPRPP-Y8U@P was few, indicating that GPRPP-Y8U@P has superb cytocompatibility toward normal cells. These results lay the necessary foundation for subsequent experiments on thrombus target therapy in vivo.

### Animal experiment

The elementary biosecurity of GPRPP-Y8U@P was demonstrated in the experiments above, and then the functional verification was carried out in rats.

**Scheme 2:**
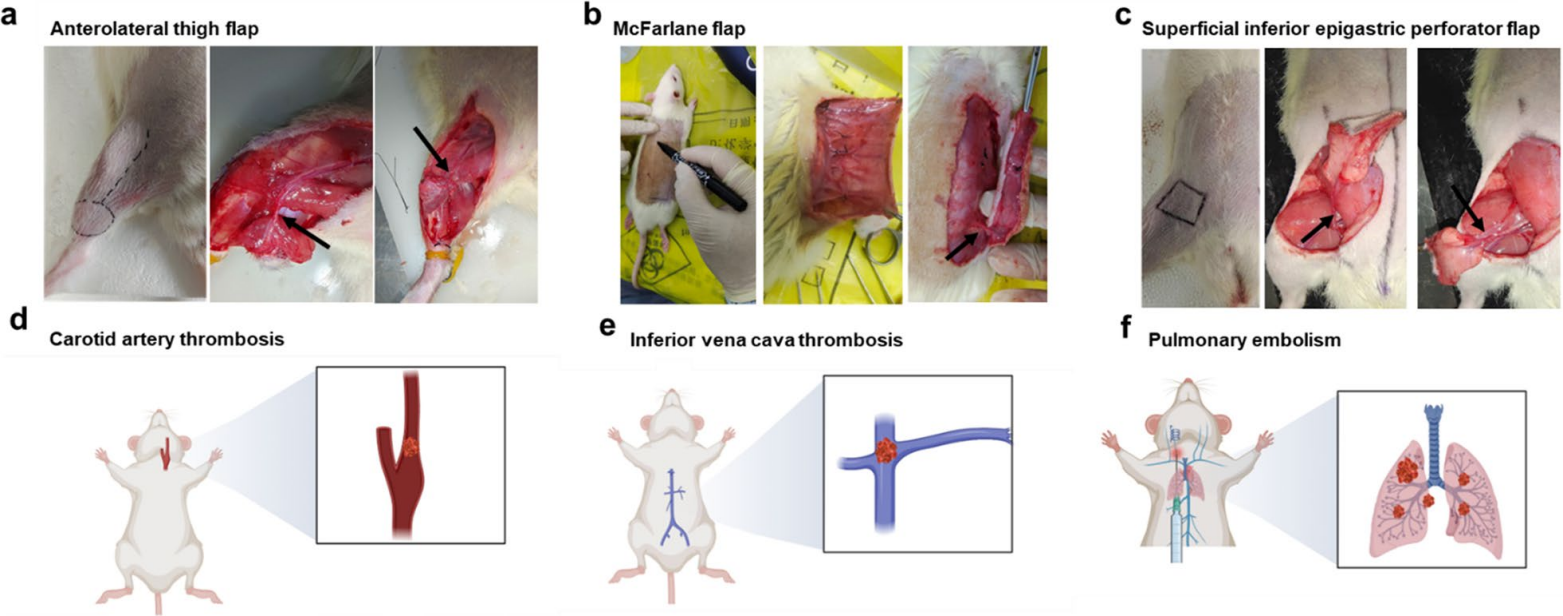
Schematic of three kind flap models and three kind thrombus animal models: an overview of methods. **a** The anterolateral thigh flap. **b** The McFarlane flap. **c** Abdominal flap: superficial inferior epigastric perforator flap. **d** Carotid artery thrombosis: topical application of filter paper saturated with 5% ferric chloride for 1 min on the left common carotid artery, corresponding vessel wall damage. **e** Inferior vena thrombosis: the inferior vena cava was ligated twice for a 4 mm length, corresponding blood stasis model. **f** Pulmonary embolism: after ligating the jugular vein's distal end, thrombin and CaCl_2_ were injected into the proximal end, corresponding blood coagulability increased

**Fig. 8:**
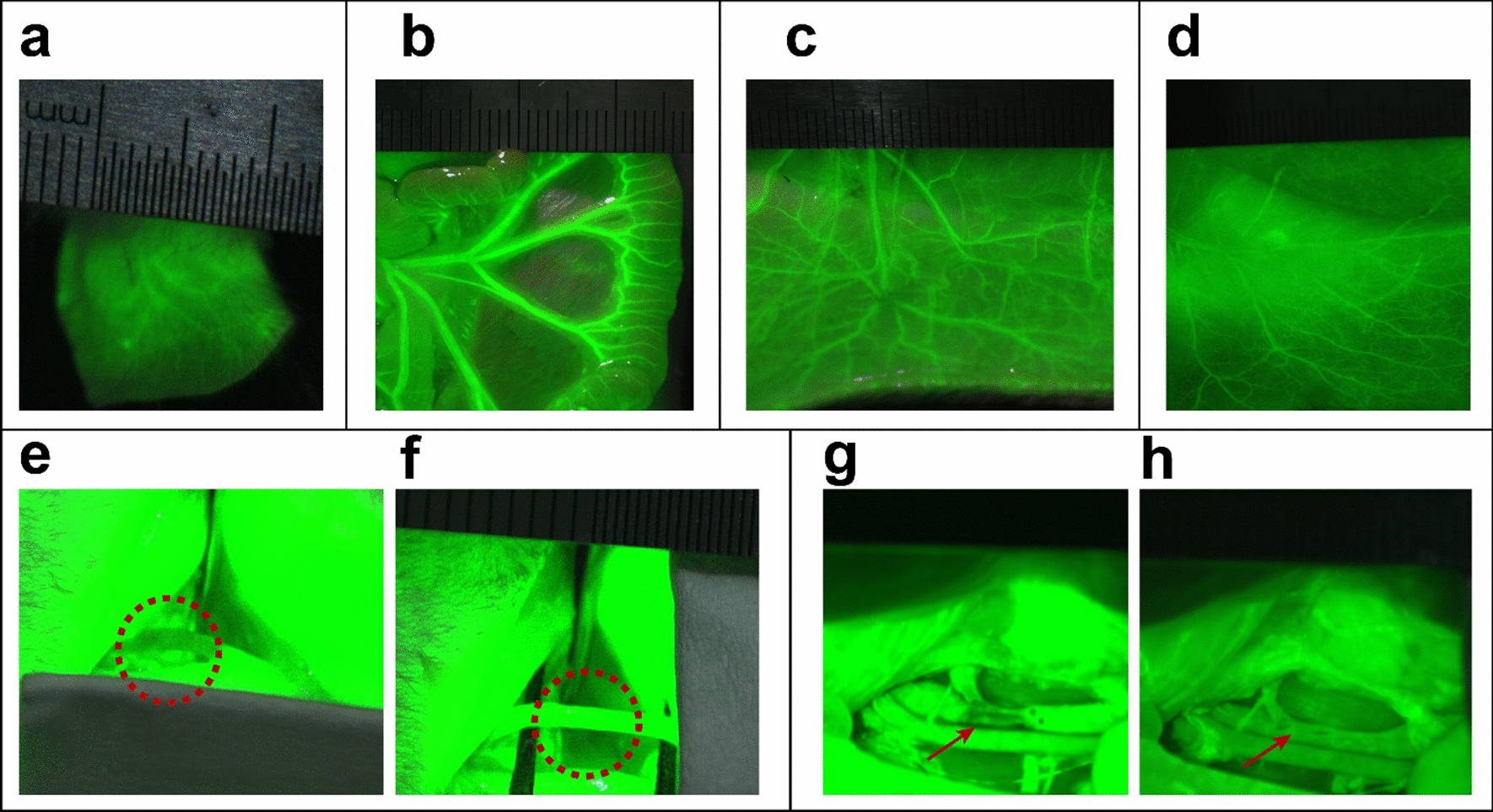
The vascular and thrombus imaging picture in vivo before and after thrombosis. **a** Vascular imaging of the ear. **b** Mesenteric artery imaging. **c** Dorsal McFarlane flap vascular imaging. **d** Vascular imaging of abdominal flap. **e**, **f** Common carotid artery imaging. The black area in the red circle shows the thrombus (left) and the image after thrombolysis (right). **g**, **h** Inferior vena cava imaging. The thrombus formed in the red circle (left) and the image after thrombolysis (right)

First, we set up the flap and general thrombus models according to Virchow's basic mechanisms of thrombosis (Scheme 2). The imaging capability was first investigated in vascular imaging. The tiny vessels of the ear, whose diameter is less than 1 mm, were seen distinctly (Fig. 8a). The mesenteric artery, McFarlane flap, and abdominal flap vascular were clearly visible (Fig. 8b–d). The fistula of the testicular can be expressly imagined, even covered by the abdominal wall muscles (Additional file 1: Fig. S5a, b), indicating the excellent tissue penetration of GPRPP-Y8U@P. After that, we also studied the thrombus monitoring ability. After topical application of filter paper saturated with 5% ferric chloride to induce thrombosis, the thrombus area was displayed by “Fluorescence extinguished”, appearing as a dark area(Fig. 8e and g). The “Fluorescence extinguished” seemed as soon as the thrombus was induced, which heralded a new era in thrombus imaging research and great potential for clinical application. After treatment with GPRPP-Y8U@P + Laser, the vascular recanalization was also reflected by the “Fluorescence recanalization”. Likewise, the flap thrombus was also detected in McFarlane and abdominal flap vascular (Additional file 1: Fig. S5 c–f). All the data proved that thrombus could be diagnosed within 5 min, consistent with the targeting results in vitro and better than most thrombus imaging agents reported in the literature [21, 41, 42]. The quick diagnosis may be due to the affinity for fibrin of GPRPP being much higher than CREKA [43]. Besides, it could also monitor vascular recanalization, meeting the clinical needs. We believed that the particles only binding to the surface of the thrombus, and the strong signal in vessels leading to the thrombus was exposed by “Fluorescence extinguished”, not “Light up”.

**Fig. 9:**
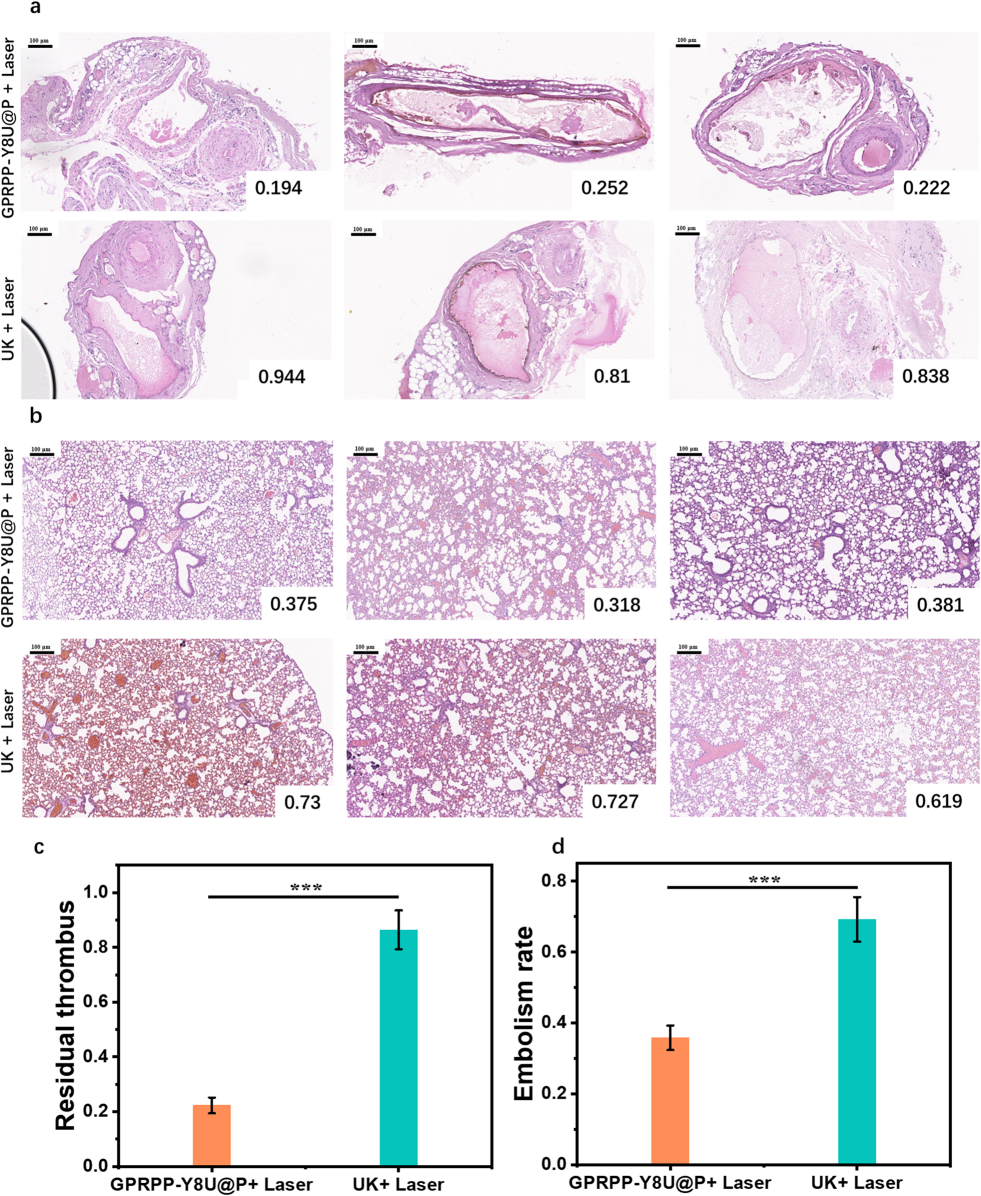
**a** HE staining of anterolateral thigh flap thrombus embolism (N = 3). The embolism rate was quantified by Image J and displayed in the lower right corner. **b** HE staining of pulmonary embolism (N = 3). The embolism rate was quantified by Image J and displayed in the lower right corner. Scale bar = 100 μm. **c** The quantitative analysis of residual thrombus in (**a**). **d** The quantitative analysis of embolism rate in (**b**). Asterisks indicate significantly differences (*P < 0.01, **P < 0.001, ***P < 0.0001)

The thrombolysis effect was assayed in two groups (the UK + Laser group and the GPRPP-Y8U@P group). For the UK + Laser group, UK (0.58 mg/40 g) was delivered by tail vein injection. Then the embolized vessels received 808 nm Laser irradiation (0.6 w/cm^2^) for 60 min. As for the pulmonary embolism animal model, the power of the Laser was increased to 1 w/cm^2^, and the irradiation area was the lung body surface projection. For the GPRPP-Y8U@P group, the rats received 1 mg/40 g (equal to 0.58 mg/40 g UK) GPRPP-Y8U@P, and the Laser was the same as the UK + Laser group. After treatment, the rats were immediately put to death, and the related vessels and lungs were removed for histological examination. The embolism rate was used to estimate the curative effect for each group, and the embolism rate was calculated as follows:

1. For the Anterolateral thigh flap thrombus embolism: the residual thrombus rate = (Embolism area/total area of the vessel) × 100% (Fig. 9a)
2. For the pulmonary embolism: random in a field-of-view under 100 × , embolism rate = (number of embolized vessels/ number of all vessels) × 100% (Fig. 9b)

Finally, we found that in the pulmonary embolism group, the embolism rate of the UK + Laser group was about 40% higher than the GPRPP-Y8U@P + Laser group. Besides, the average rate in the anterolateral thigh flap model was nearly 60% higher (Fig. 9). Compared with the UK alone, the thrombolytic efficiency, containing thrombolytic time and residual thrombus, improved significantly after being combined with PDT and PTT. The thrombus can be reduced by about 50% within 60 min, which meets the urgent need to improve hemodynamics in most thrombotic events. In particular, these results were obtained at relatively low laser power, giving rise to biosecurity.

In a nutshell, we brilliantly testified the thrombus imaging ability and thrombolysis effectiveness of GPRPP-Y8U@P in this section. We believed that GPRPP-Y8U@P could remarkably realize thrombus targeting and treatment with quick diagnosis and vascular recanalization capacity.

### Evaluation of safety in vivo

**Fig. 10:**
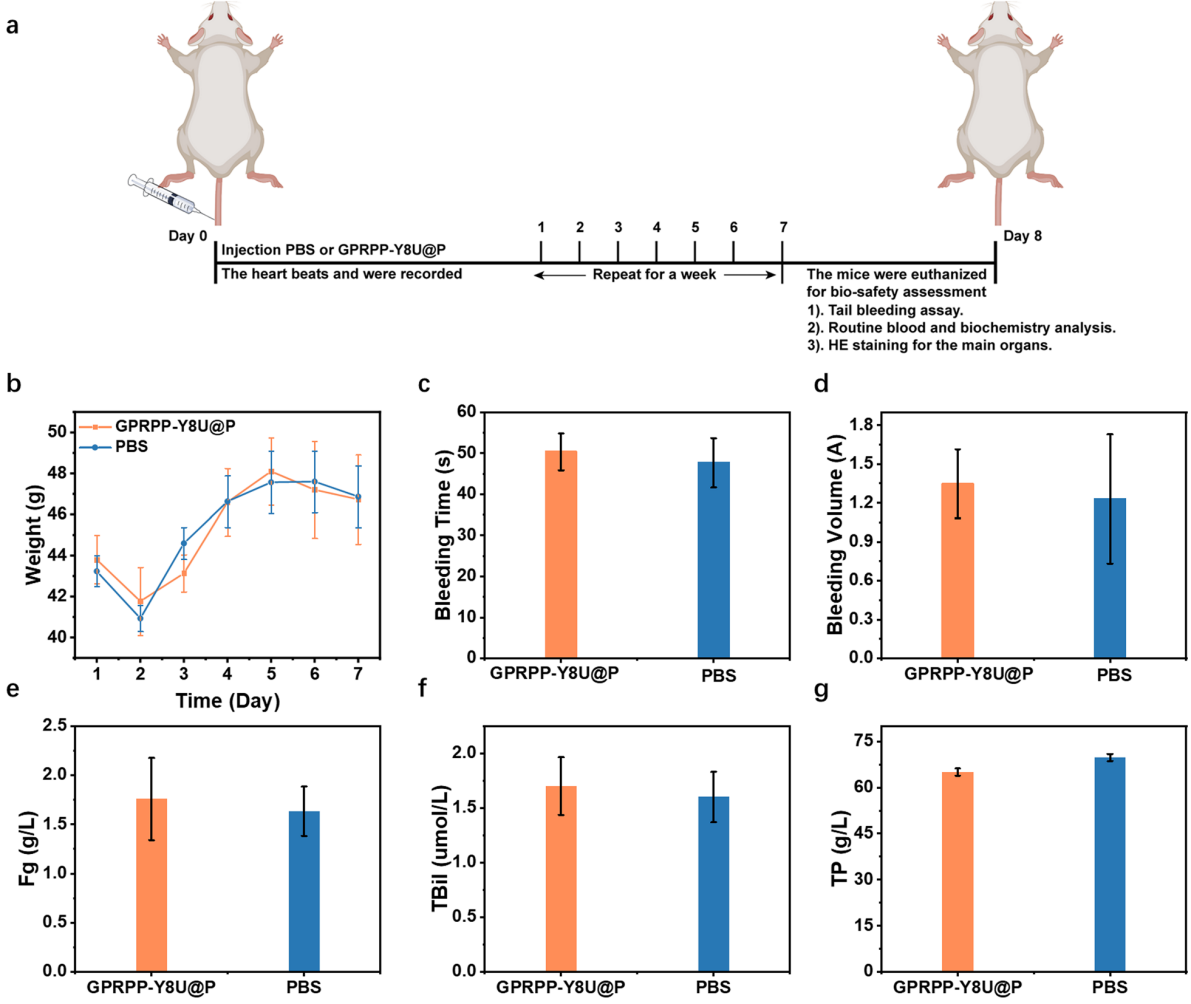
**a** Schematic of the security assessment process in vivo. PBS (100 μL) or GPRPP-Y8U@P (1 mg/mL, 100 μL) was administrated by tail vein injection for a week. During this period, the weight change were recorded to reflect the impact of hematologic toxicity. Afterward, a tail bleeding assay was performed to assess the model mice’ hemostatic function and bleeding risk. **b** The daily change of weight. **c**, **d** The bleeding time and volume in the tail bleeding assay. **e**–**g** Some biochemical indicators (Fg: fibrinogen, TBil: total bilirubin, TP: total protein)

**Fig. 11:**
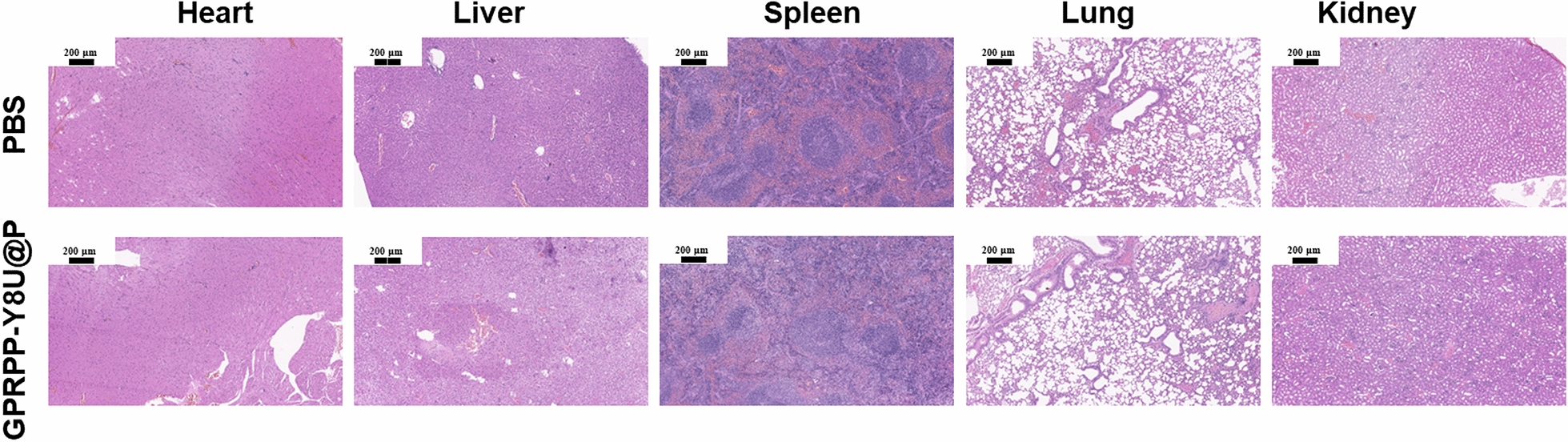
HE staining of main organs. Scale bar = 100 μm

**Fig. 12:**
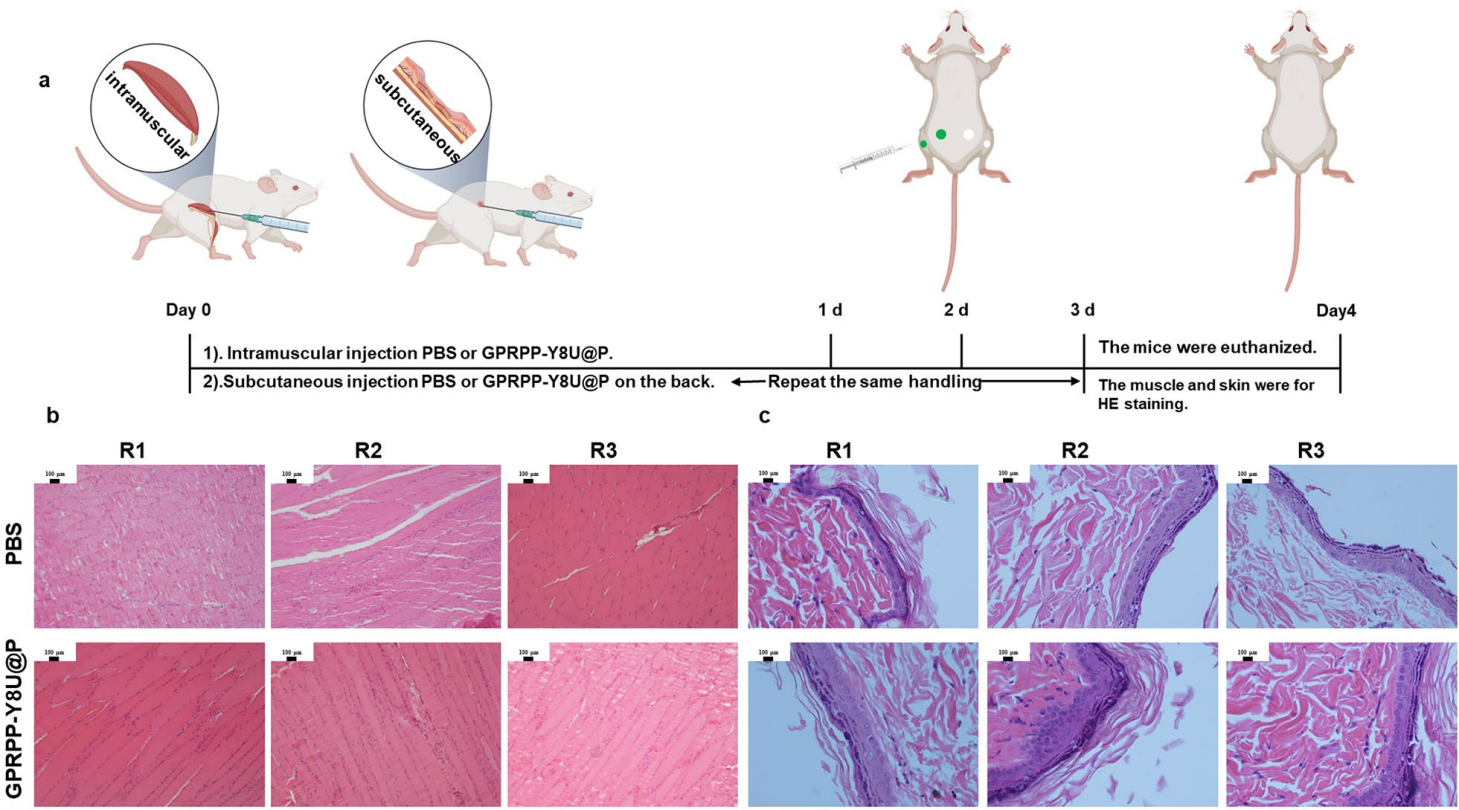
**a** Schematic of the skin and muscle sensitization assay. The mice were subcutaneous and intramuscular injected with PBS (100 μL) or GPRPP-Y8U@P (1 mg/mL, 100 μL) once a day. After 3 days, the injection sites were for HE staining to evaluate sensitization. **b** The HE staining of muscle over the injection site. **c** The HE staining of skin over the injection site. R1, R2, and R3 represented repeat groups

The biosafety assessment in vivo was performed as in Fig. 10a. First, the KM mice received an injection of PBS (100 μL) or GPRPP-Y8U@P (1 mg/mL, 100 μL). The weight change was recorded for a week with no significant differences (Fig. 10b). After that, the tails were cut for tail bleeding assay, and we measured the bleeding time and volume, which were in the normal range compared with the control group (Fig. 10c, d). Then, the mice were euthanized, and the blood was collected for routine blood and biochemistry analysis. Based on these indicators, the biosafety of GPRPP-Y8U@P was identified (Fig. 10e–g). Finally, the HE sections of the main organs were used for safety assessment after the formalin-fixed and paraffin-embedded tissue section. No organ tissue lesions were observed from HE sections (Fig. 11). The above data analysis fully proved that no constitutional signs or toxicities caused by GPRPP-Y8U@P were observed, at least during the entire experimental period. The skin and muscle sensitization assay were also evaluated by HE sections and carried out, as shown in Fig. 12a. One side of each mouse's back received subcutaneous PBS and intramuscular PBS in the leg. Then, the other side received a GPRPP-Y8U@P injection for self-control. After three consecutive days, skin and muscle were excised over the injection site for HE staining. No signs of tissue damage were demonstrated again. In conclusion, the biosafety assessment observed no behavioral, weight loss, or poisoning abnormalities. GPRPP-Y8U@P has good histocompatibility and can be used as thrombus-targeted therapy material.

### Drug distribution and metabolism

The rats' organs and blood were collected to measure the light intensity emitted by GPRPP-Y8U@P. As shown in Additional file 1: Fig. S6, the particles mainly existed in the liver and spleen. Almost all the particles were cleared after 5 days in the liver and 8 h in the spleen. The reticular-endothelial system filtrated the particles, leading to the particles mainly existing in the liver and spleen. Besides, the intensity in the blood could keep significant for nearly 24 h. This data showed that GPRPP-Y8U@P had the potential to meet clinical needs since its cycle time covered the surgery and the first night after surgery.

## Conclusions

In summary, a developed thrombus integrated nanomedicine GPRPP-Y8U@P for fibrin-targeting and antithrombotic therapy. We first focused on flap thrombus-targeted treatment and achieved an excellent curative effect. Based on the fibrin-targeting ability of GPRPP and the NIR-II imaging effect of Y8 NPs, GPRPP-Y8U@P can place the thrombus area intuitively at once. NIR-­II imaging-guided UK/PDT/ PTT therapy was accomplished on the thrombus animal model to abate thrombus under NIR laser irradiation. The appropriateness of GPRPP-Y8U@P for thrombus treatment has been illustrated by the high effect of thrombolysis and shallow risk of bleeding. Therefore, our work accomplishes flap visualizing monitoring and focuses on flap thrombolysis in situ for the first time. Overall, our present work on the well-designed principle and thrombus therapy of GPRPP-Y8U@P is a potent strategy for antithrombotic therapy and flap rescuing. In-depth studies in the future may significantly improve the flap and thrombus monitoring efficiency.

## Materials and methods

### Materials

Poly (lactic-co-glycolic acid) (PLGA; containing 75% lactide and 25% glycolide, with a molecular weight of 15,000, end group = COOH) was purchased from Daigang Biological Material (Shangdong, China). GPRPP (Gly–Pro–Arg–Pro–Pro) peptides for Fibrin-targeting were made by Jier Biological (shanghai, China). Organic Near-Infrared-II Nanophotosensitizer Y8 was purchased from Derthon (Shenzhen, China). Urokinase plasminogen activators (UK) from Bioss (Beijing, China). Thrombin and fibrinogen were obtained from Senbeijia (Nanjing, China) and Solarbio(Beijing, China). Dichloromethane (DCM), Polyvinyl alcohol (PVA), and FeCl_3_ were purchased from Admas-Beta (Shanghai, China). Ethane sulfonic acid (MES), N-hydroxysuccinimide (NHS), and 1-ethyl-3-(3-dimethyl aminopropyl) carbodiimide hydrochloride (EDC) were purchased from Sigma-Aldrich Corporation (St Louis, MO, USA). SOSG kit was purchased from Invitrogen (Thermo Fisher Scientific, USA), DAPI, LDH Cytotoxicity Assay Kit, Calcein-AM/PI Double Stain Kit, and BCA Protein Assay Kit were obtained from Beyotime (Shanghai, China). Annexin V-FITC/PI apoptosis detection kit was obtained from Vazyme (Nanjing, China). Fetal bovine serum (FBS) was purchased from Biological Industries (Israel), and Cell Counting Kit-8 (CCK-8) was purchased from Bimake (Shanghai, China).

### Preparation of Y8U@P and Y8@P nanoparticles

A classical double emulsification process was used to prepare the NPs [44–46]. Briefly, 1 mL 0.1% PVA was added to 10 mL Dichloromethane with 100 mg PLGA and 10 mg Y8. After ultrasonic emulsification with 100 W (SM-650D, Shunmayq, Nanjing, China), a water-in-oil (w/o) emulsion is formed. Then, this w/o emulsion was added to 50 mL 2% PVA and acoustically emulsified for the same time to form w/o/w emulsion. Finally, The w/o/w emulsion was kept for 4 h in an ice bath under gentle stirring until the organic solvents were volatilized entirely. The Y8 NPs (Y8@P) were collected by centrifugation and washed several times with PBS. As for the Y8-UK NPs (Y8U@P), the 0.1% PVA with the UK was used to replace the pure 0.1% PVA. The rest of the steps are the same as the Y8 NPs. Besides, for the convenient visualization of NPs in several experiments under the confocal laser scanning microscope, using Ce6 in DMSO replaced the UK in 0.1% PVA, we synthesized Y8-Ce6 NPs (Y8C@P).

### The loading of targeting group

EDC/NHS was added at a molar ratio of 1:2 to 0.1 M MES buffer solution (pH = 5.5) to activate the carboxyl of the NPs synthesized above on a plate shaker at 4℃ for 1 h. (The EDC: carboxyl was at a molar ratio of 10:1). Then, centrifugal removal of unreacted EDC/NHS and the activated NPs were resuspensions with 0.1 M MES buffer solution (pH = 8.5), and 20 mg GPRPP peptide was added. Incubation was performed at 4 °C overnight on a plate shaker. Finally, in the same way, the GPRPP-Y8U@P NPs were obtained from Y8U@P via centrifugal and washing. GPRPP-Y8@P and GPRPP-Y8C@P were synthesized from Y8@P NPs and Y8C@P NPs, respectively. Through freeze-dried, the NPs powder was acquired and ready for use.

### Characterization of GPRPP-Y8U@P

FTIR was used to prove the successful loading of GPRPP on the surface of nanoparticles compared to Y8@P and GPRPP-Y8@P. The protein was quantified using the BCA Protein Assay. The size and morphology of different nano-drug were characterized by TEM (JEOL JEM 2100plus) and DLS (Dandong BT-90 Liaoning). The ultraviolet–visible (UV–vis) spectrum absorption spectrum was obtained by a UV–vis–NIR spectrophotometer (PerkinElmer LAMBDA 365), and the fluorescence spectrum was obtained by a photoluminescence spectrometer (FLS920, fluorescence spectrometer, Edinburgh Instruments, UK). The absorption coefficients of GPRPP-Y8U@P were also calculated. The GPRPP-Y8U@P was dispersed in DMEM and PBS (pH = 5.4, pH = 7, pH = 8.4) and stored for 7 days to evaluate the storage stability.

1. The yield of Y8U@P
  Y8U@P freeze-dried powder was obtained after drying in a vacuum oven at room temperature overnight. The yield was calculated by: yield = dry power weight / (PLGA weight + Y8 weight + UK weight) × 100%.
2. Y8 loading ratio
  The dry powder was dissolved in DCM, and the absorbance at 600 nm was recorded. The standard curve of Y8 calculated the loading ratio in DCM.
3. UK loading ratio
  The dry powder was dissolved in DCM and diluted by PBS (1:10). Then, the UK was quantified by BCA Protein Assay Kit.
4. GPRPP loading ratio
  During synthesizing GPRPP-Y8@P, after reacting with GPRPP, the supernatant of the first centrifuge was used to quantitative the unreacted GPRPP. The GPRPP loading ratio = (whole GPRPP—GPRPP in the supernatant)/ whole GPRPP.
5. Absorption coefficients of GPRPP-Y8U@P
  The absorbance of different concertation GPRPP-Y8U@P at 600 nm was recorded, and linear curve fitting was performed. The absorption coefficient was the slope of the fitting equation.

### Photodynamic Performance of GPRPP-Y8U@P

GPRPP-Y8U@P (10 μg/mL, PBS) was mixed with SOSG (5 μM) and irradiated by Laser (808 nm, 1.0 W/cm^2^). The fluorescence spectrum of oxidized SOSG (excitation/emission: 488/525 nm) was recorded by a fluorescence spectrophotometer (FL6500, Shanghai, China) every 10 s.

### Photothermal performance of GPRPP-Y8U@P

The photothermal performance of GPRPP-Y8U@P was measured by recording the GPRPP-Y8U@P solution (1, 0.5, and 0.25 mg/mL) temperature change with 808 nm laser irradiation (1.2, 0.6, and 0.3 W/cm^2^) by a thermometer (C3, FLIR, USA). The size of nanoparticles and heating–cooling curve for several cycles were evaluated for the Photothermal cycle stability.

### Drug release

To evaluate NIR light-triggered UK release, 5 mg of Y8U@P was dispersed in 5 ml of PBS buffer and exposed to 808 nm near-infrared (0 and 0.6 W/cm^2^) for 2 h. The power was the same as in vivo experiment. Supernatants were collected every 10 min, and UK release was measured with a BCA kit.

### NIR-II fluorescence imaging and tissue penetration depth

The NIR-II Series III 900/1700 In Vivo Imaging System (Suzhou NIR-Optics Co., Ltd., China) imagined different concentrations of GPRPP-Y8U@P in the tube. As for tissue penetration depth, 0.1 mg/mL GPRPP-Y8U@P was put into a capillary tube with a 1 mm diameter. Then, they were placed under agar blocks with different thicknesses of 2 mm, 4 mm, 6 mm, 8 mm, and 10 mm. And then, they were placed under fresh chicken breast muscle, during which the signal-to-noise ratio (SNR) and full width at half-maximum (FWHM) were calculated.

The SNR means that the tube signal above background noise quantitatively and calculated as follows [47]:

$${\text{SNR}} = \left( {{\text{signal}}\,{\text{intensity}}\,{\text{in}}\,{\text{the}}\,{\text{tube}}} \right)/\left( {{\text{signal}}\,{\text{intensity}}\,{\text{background}}} \right).$$

The FWHM value was calculated as the width of the peak at the half-maximal intensity and obtained by the Gaussian fitting in ImageJ-win64 to evaluate the spatial resolution of GPRPP-Y8U@P [48].

### Preparation of artificial thrombus

In this part, we prepared several kinds of artificial thrombus. First, for the thrombolysis evaluation in vitro, fresh blood collected from the rat abdominal aorta was mixed with thrombin (500 U/mL) and CaCl_2_ (10% w/v), then placed into a water bath at 37 °C for 2 h. The above thrombus was placed at 4℃ for a week to prepare the old thrombus. For the white (platelet-rich) thrombus and red (red blood cell-rich) thrombus [38], fresh blood was centrifugated to obtain red blood cells and platelets, then mixed at the ratio of 9:1 or 1:9. Afterward, we added thrombin and CaCl_2_ to form a red and white thrombus.

### Targeting ability in vitro

To verify the binding ability of GPRPP-Y8U@P, FITC-fibrinogen and GPRPP-Y8C@P were prepared for convenient visualization and capture by confocal microscope [49, 50]. Fibrinogen was incubated with FITC in PBS for 4 h, and then dialysis was employed to remove free FITC. After that, fibrin clots were formed on the small petri dish. 200 μL of fibrinogen, 20 μL of CaCl_2_ (0.2 M/L), and 2 μL of thrombin (100 U/mL) were added and incubated for 3 h at 37 ℃. Finally, these dishes were randomized into the GPRPP-Y8C@P group, the Y8C@P group, and the GPRPP + GPRPP-Y8C@P group and co-cultivated for 15 min. Then they were washed three times by PBS and observed under Confocal Microscope. Under NIR-II In Vivo Imaging System, the thrombus samples were randomized into three groups: the GPRPP-Y8U@P group, the Y8U@P group, the GPRPP + GPRPP-Y8U@P group, and the PBS group.

Among all groups, the thrombus was incubated with different materials for 15 min and washed three times with PBS. As for the GPRPP + GPRPP-Y8U@P or GRPPP + GPRPP-Y8C@P group, the thrombus was first incubated with GPRPP for 15 min and set with GPRPP-Y8U@P or GPRPP-Y8C@P for 15 min, then washed by PBS. Similarly, the red, white, and old thrombus were randomized into three groups: the GPRPP-Y8U@P group, the Y8U@P group, and the GPRPP + GPRPP-Y8U@P group. After incubating for 5 min, the signal intensity was checked. Moreover, GPRPP-Y8U@P was incubated with a thrombus under a static state to evaluate how long the nanoparticles needed to bind the thrombus.

### In vitro assessment of thrombolysis activity

The thrombolysis activity was evaluated by weight loss ratio after treatment. Above all, artificial thrombus from SD rats was cut into equal blocks and weighted, marked as original weight. The thrombus was placed in glass bottles for the static thrombolysis and randomly divided into four groups (n = 3 per group): the UK group, the UK + Laser group, the GPRPP-Y8U@P group, and the GPRPP-Y8U@P + Laser group. The concentration of GPRPP-Y8U@P was 1 mg/mL. The treating time was set as 30 min. After treatment, filter paper removed surface moisture, and the thrombus was weighed again and marked as the final weight. Considering blood flowing in vivo, we assembled a simulated extracorporeal circulation model with a peristaltic pump, beaker, 5 mL centrifuge tube, and laser device. The grouping and treating time adopted was the same as the static thrombolysis, and the thrombolysis rate was calculated finally.

The thrombolysis activity was calculated as follows: thrombolysis rate (thrombolysis activity) = (original weight -final weight)/(initial weight) × 100%.

### Blood compatibility evaluation

#### Platelet adhesion

The degree of platelet damage caused by GPRPP-Y8U@P was determined by measuring the amount of lactate dehydrogenase (LDH) by the LDH activity kit (Beyotime Biotechnology) to the manufacturer's protocol [51]. Briefly, 600 μL of rat fresh platelet-rich plasma (PRP) from fresh anticoagulant blood was placed on the GPRPP-Y8U@P tube (1 mg in 100 μL PBS), PBS (100 μL), and glass samples (1 mg in 100 μL PBS). All samples were incubated at 37 °C for 60 min. The suspension was obtained via centrifugal, transferred to a 96-well plate, and detected following the manufacturer's protocol.

#### Hemolysis assay

Red blood cell was extracted from fresh rat whole blood and mixed with PBS to prepare 2% red blood cell suspension, then incubated with PBS, 0.1%Triton-100, and GPRPP-Y8U@P solution at different concentrations for 3 h to confirm the blood compatibility [52]. Finally, the absorbance of the supernatant was determined at 540 nm using a microplate reader, reflecting the hemoglobin from lysed red blood cells. PBS and 0.1%Triton-100 were used as a negative and positive group, respectively [52]. Then each tube was centrifuged at 1000 g for 12 min. The absorbance of the supernatant can reflect hemolysis and directly indicate the relative damage of different treatments to erythrocytes. The hemolysis ratio can also be calculated as follow: Hemolysis (%) = (GPRPP-Y8U@P absorbance-negative group)/(positive group- negative group) × 100%.

#### Plasma recalcification time (PRT)

Platelet-rich plasma was first centrifuged from fresh rat blood anticoagulated with sodium citrate after activating by 200 μL CaCl_2_ and adding into a glass bottle with nanoparticles or thrombin as positive control or PBS as a negative control at 37℃. Monitoring while shaking until the time of sample solidification was recorded as PRT [53].

### Cytotoxicity test

Human umbilical vein endothelial cells (HUVEC) and rat macrophages (RAW264.7) were cultured in DMEM High Glucose medium containing 100 U/mL of penicillin, 100 μg/mL streptomycin, and 10% fetal bovine serum at 37 °C in a 5% CO_2_ environment. The cells were seeded into a 96-well plate, a 24-well plate, and a 6-well plate for microplate reader, fluorescence microscope, and flow cytometric detection. After being incubated with GPRPP-Y8U@P for 24 h, the simple co-incubation group was directly used for detection. A 5-min 808 nm laser was performed in the laser co-incubation group with different power before detection. Finally, the cytotoxicity of GPRPP-Y8U@P in HUVEC and RAW264.7 was detected by the CCK-8 assay, Calcein-AM/PI Double Stain Kit, and Annexin V-FITC/PI apoptosis detection kit. For the CCK-8 assay, the cell viability(%) was reflected by the absorbance at 450 nm. Then, the cell viability was measured following the CCK-8 assay, and the live and dead cells were distinguished by Calcein-AM/PI Double Stain Kit and Annexin V-FITC/PI apoptosis detection kit.

### Animal experiment

KM mice (30–40 g) and SD rats (200–300 g) were purchased from Vital River Animal Laboratories and maintained in SPF level animal room at 22 °C under 12 h light–dark cycle and food and water ad libitum. All animal experiments were approved by the Institutional Animal Care and Use Committee of Nanjing Stomatological Hospital Medical School of Nanjing University.

#### Thrombus model (pulmonary embolism, carotid artery thrombosis, and inferior vena cava thrombosis model)

SD ratse (200–300 g) were first anesthetized with 20% urethane (0.5 mL/100 g) to establish these mice models. For pulmonary embolism, the right internal jugular vein was separated. After ligating the distal end of the jugular vein, thrombin(100 U/kg) in 2% CaCl_2_ was injected into the proximal end of the ligation. An electric heating blanket maintained the mouse body at around 37 °C. After careful hemostasis, the ligation was removed, and any dead mice were abandoned. For the carotid artery thrombosis model, the skin was incised in the middle of the neck, and the left common carotid artery was exposed completely following blunt muscle separation. Then, topical application of 3*3 mm filter paper is saturated with 5% ferric chloride (FeCl_3_) for 1 min on the left common carotid artery to induce blood clots. Finally, the abdominal cavity was entered and exposed via a median incision on the abdominal wall for the inferior vena cava thrombosis model. Whole intestines were pulled aside and covered with saturated 0.9% NaCl gauze. Then, the inferior vena cava was freed and ligated twice for about 3 mm length. The inferior vena cava model was set up 6 h after closing the abdomen layer-to-layer with 6–0 non-absorbable sutures. Before the experiment, we reopened the abdomen and removed the ligation so that nanoparticles could reach the thrombus.

#### Anterolateral thigh flap, McFarlane flap, and superficial inferior epigastric perforator flap animal model

To establish the skin flap animal model, SD rats (200–300 g) received 20% urethane (0.5 mL/100 g) for anaesthetization, freeing out the arteriasaphena and great saphenous following the operation mark, and then cut off the skin flap supplied by the vascular network at the end of arteriasaphena. As for the other flap models, the steps were similar. In brief, free the setting blood vessels and cut off the flap supported by the vessel. Then, suitable size filter paper saturated with 5% ferric chloride was applied for a while on the related vessel to construct the thrombus skin flap model.

### Biosafety assessment of GPRPP-Y8U@P in vivo

#### Systemic chronic toxicity assay

Six KM mice (30–40 g) were randomly divided into two groups. The two groups were injected with a therapeutic dose of GPRPP-Y8U@P or the same dose of PBS daily for a week. During this period, the weight, heartbeats, and toxic symptoms were recorded every morning at 10:00 a.m. On the eighth day, the KM mice first performed a tail bleeding assay was reported [42]. The tail was briefly cut off to induce bleeding, and the bleeding time and volume were recorded on the seventh day. The bleeding time was defined as the time to complete the cessation of bleeding for 15 s. The bleeding volume was collected in PBS at 37 ℃ and quantified by the absorbance at 450 nm. Afterward, the KM mice were euthanized. The blood and main organs were collected for blood routine (urea, creatinine, glutamic-pyruvic transaminase, aspartate aminotransferase, etc.), coagulation indicators, and H&E staining, respectively.

#### Skin and muscle sensitivity test

In this part, six KM mice (30–40 g) were randomly divided into two groups and injected PBS or GPRPP-Y8U@P subcutaneously on the back and intramuscular in the gluteus maximus muscle. After 3 days, the KM mice were euthanized, and the skin and muscles were excised for H&E staining.

#### Drug distribution and metabolism

Six rats (200–300 g) were injected with 1 mL GPRPP-Y8U@P (1 mg/mL) and collected organs and blood at time points. The drug distribution and metabolism were studied by measuring the intensity of the fluorescence of organs and blood.

## Supplementary Information

**Additional file 1:** **Figure S1.** The Molecular Structure of Y8. **Figure** **S2.** (a) The TEM image and DLS of Y8U@P. (b) The standard curve of Y8 in DCM at 600 nm. (c) The absorption spectrum of GPRPP-Y8U@P in DCM. (d) The BCA standard curve in the supernatant to calculate the UK loading rate. **Figure S3.** The cumulative release of UK under NIR irradiation (0 and 0.6 w/cm2). **Figure S4. **(a) Raw264.7 viability after simple co-incubation with GPRPP-Y8U@P by fluorescence microscopy. (b) Raw264.7 viability after simple co-incubation with GPRPP-Y8U@P by a microplate reader. (c) Raw264.7 viability after laser co-incubation with GPRPP-Y8U@P by fluorescence microscopy. (d) Raw264.7 viability after laser co-incubation with GPRPP-Y8U@P by a microplate reader. (e) Apoptosis of Raw264.7 under co-incubation with GPRPP-Y8U@P at different concentrations. (d) Apoptosis of Raw264.7 under laser co-incubation with GPRPP-Y8U@P at different powers. **Figure S5**. The vascular and thrombus imaging picture in vivo before and after thrombosis. (a) - (b) Fistula of testicular imaging under abdominal wall muscles (a) or exposed to air (b). (c) - (d) Vascular imaging of abdominal flap donor by superficial epigastric arteriovenous, and the black area in the red circle means the thrombus. (e) - (f) Dorsal McFarlane flap vascular imaging and the black area in the red circle represented thrombus. **Figure S6.** (a) Distribution of nanoparticles in organs. (b) Quantitative statistics in (a). (c) Organ metabolism. (d) Blood metabolism. Group 1 in (c) and (d) represents the blank group, 2-8 represent 2h, 4h, 8h, 16h, 1d, 3d, and 5d after injection.

## Data Availability

The data supporting this study's findings are available within the paper and its Supplementary Information.
