# Supplementary material for: Thrombus-targeted nano-agents for NIR-II diagnostic fluorescence imaging-guided flap thromboembolism multi-model therapy
Source: J Nanobiotechnology. 2022 Oct 14;20:447. doi: 10.1186/s12951-022-01649-6 (PMC9563174; doi:10.1186/s12951-022-01649-6)
Supplement: Supplementary file 1 — Additional file 1: Figure S1. The Molecular Structure of Y8. Figure S2. (a) The TEM image and DLS of Y8U@P. (b) The standard curve of Y8 in DCM at 600 nm. (c) The absorption spectrum of GPRPP-Y8U@P in DCM. (d) The BCA standard curve in the supernatant to calculate the UK loading rate. Figure S3. The cumulative release of UK under NIR irradiation (0 and 0.6 w/cm2). Figure S4. (a) Raw264.7 viability after simple co-incubation with GPRPP-Y8U@P by fluorescence microscopy. (b) Raw264.7 viability after simple co-incubation with GPRPP-Y8U@P by a microplate reader. (c) Raw264.7 viability after laser co-incubation with GPRPP-Y8U@P by fluorescence microscopy. (d) Raw264.7 viability after laser co-incubation with GPRPP-Y8U@P by a microplate reader. (e) Apoptosis of Raw264.7 under co-incubation with GPRPP-Y8U@P at different concentrations. (d) Apoptosis of Raw264.7 under laser co-incubation with GPRPP-Y8U@P at different powers. Figure S5. The vascular and thrombus imaging picture in vivo before and after thrombosis. (a) - (b) Fistula of testicular imaging under abdominal wall muscles (a) or exposed to air (b). (c) - (d) Vascular imaging of abdominal flap donor by superficial epigastric arteriovenous, and the black area in the red circle means the thrombus. (e) - (f) Dorsal McFarlane flap vascular imaging and the black area in the red circle represented thrombus. Figure S6. (a) Distribution of nanoparticles in organs. (b) Quantitative statistics in (a). (c) Organ metabolism. (d) Blood metabolism. Group 1 in (c) and (d) represents the blank group, 2-8 represent 2h, 4h, 8h, 16h, 1d, 3d, and 5d after injection. [file 12951_2022_1649_MOESM1_ESM.docx]

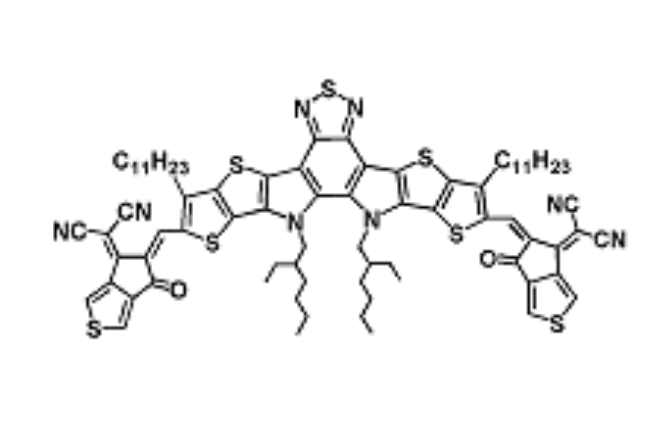


**Figure S1.** The Molecular Structure of Y8


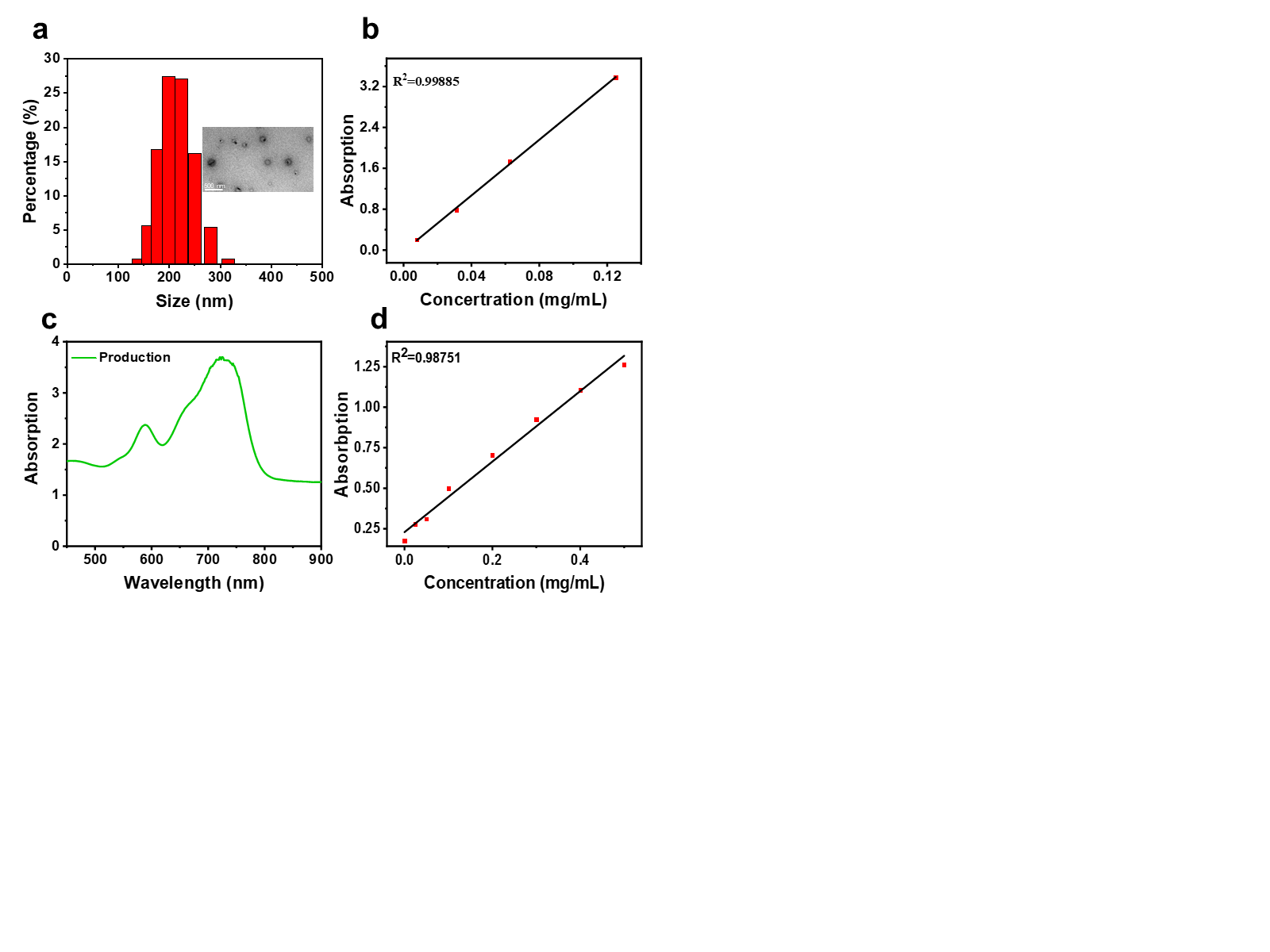


**Figure** **S2.** (a) The TEM image and DLS of Y8U@P. (b) The standard curve of Y8 in DCM at 600 nm. (c) The absorption spectrum of GPRPP-Y8U@P in DCM. (d) The BCA standard curve in the supernatant to calculate the UK loading rate.


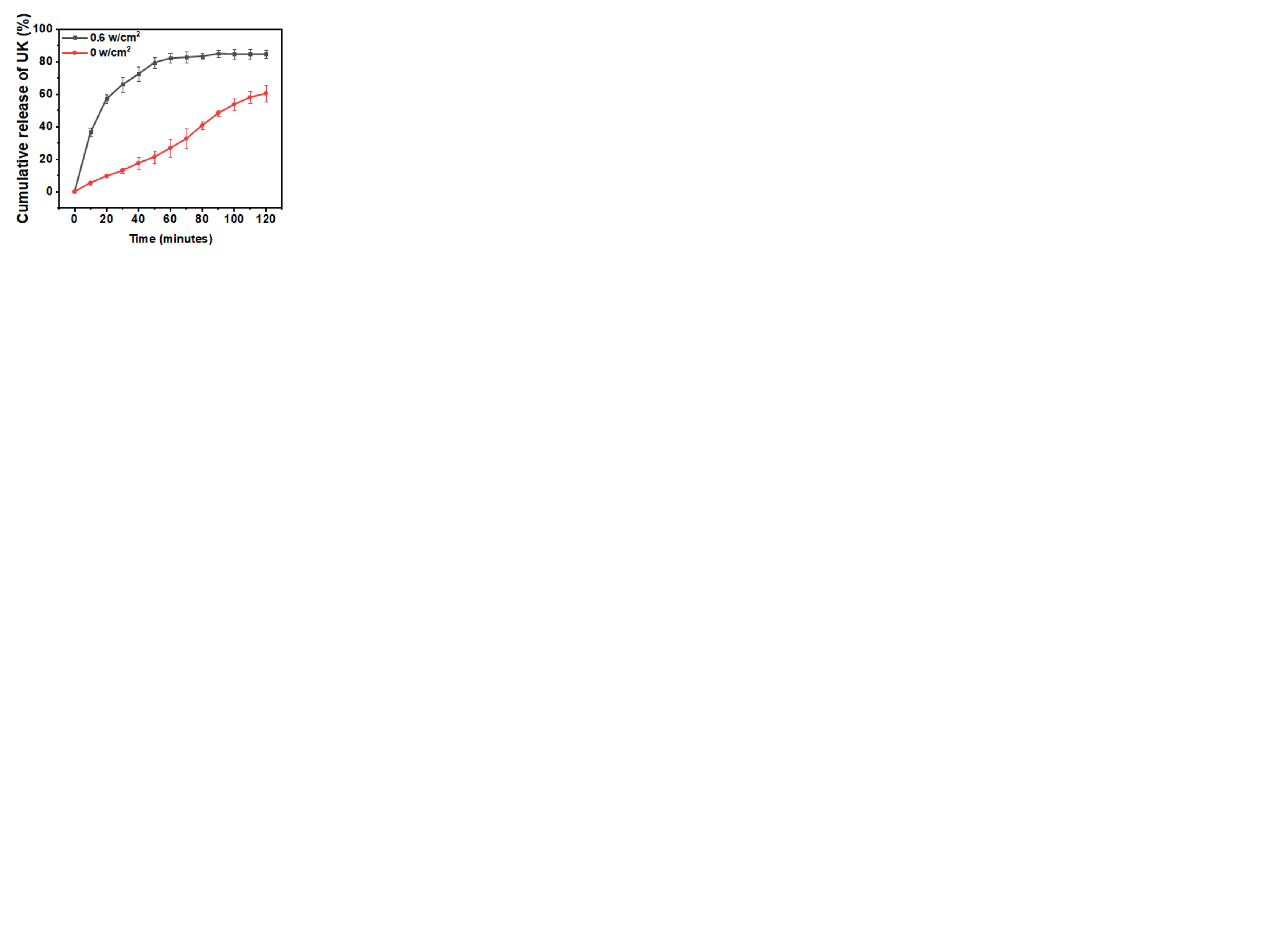


**Figure S3.** The cumulative release of UK under NIR irradiation (0 and 0.6 w/cm2).


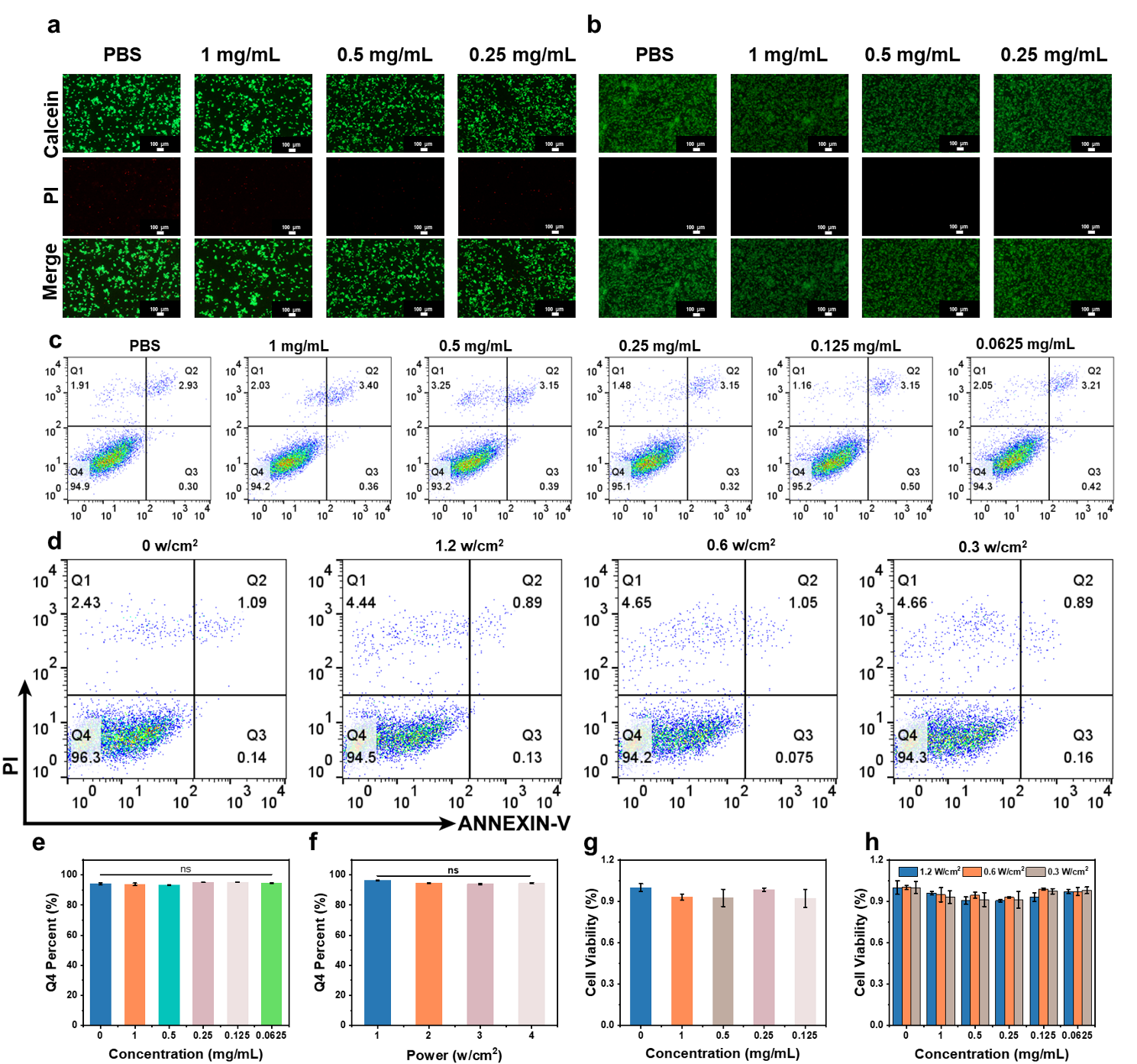


**Figure S4.** (a) Raw264.7 viability after simple co-incubation with GPRPP-Y8U@P by fluorescence microscopy. (b) Raw264.7 viability after simple co-incubation with GPRPP-Y8U@P by a microplate reader. (c) Raw264.7 viability after laser co-incubation with GPRPP-Y8U@P by fluorescence microscopy. (d) Raw264.7 viability after laser co-incubation with GPRPP-Y8U@P by a microplate reader. (e) Apoptosis of Raw264.7 under co-incubation with GPRPP-Y8U@P at different concentrations. (d) Apoptosis of Raw264.7 under laser co-incubation with GPRPP-Y8U@P at different powers.


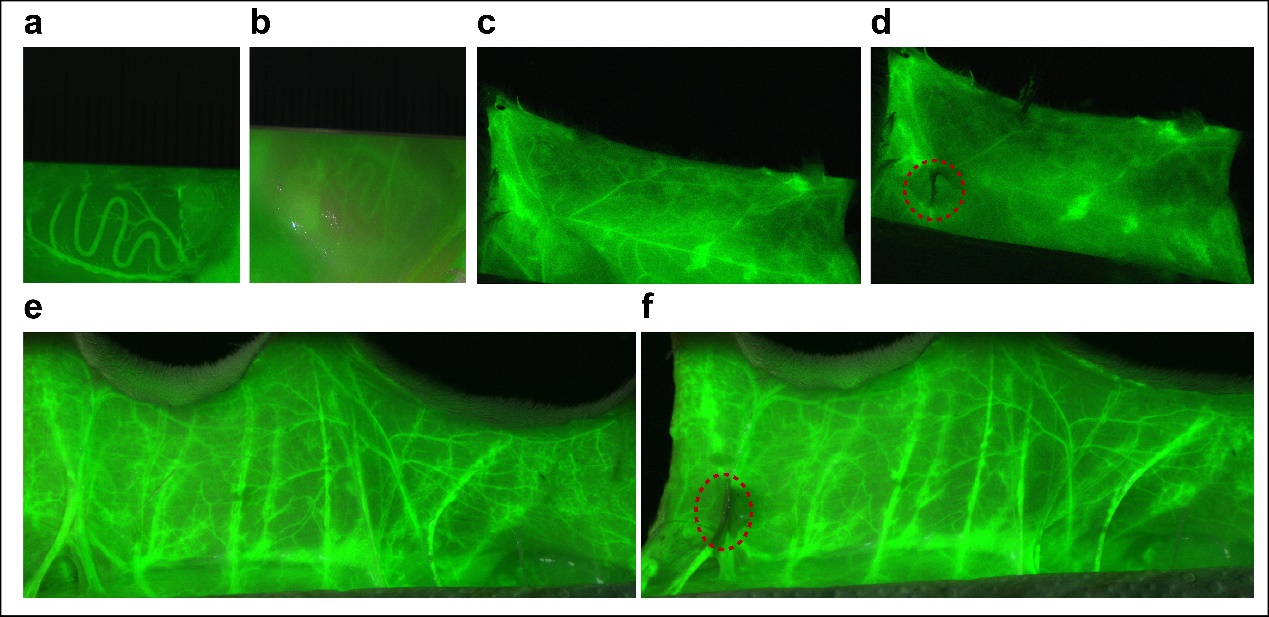


**Figure S5**. The vascular and thrombus imaging picture in vivo before and after thrombosis. (a) - (b) Fistula of testicular imaging under abdominal wall muscles (a) or exposed to air (b). (c) - (d) Vascular imaging of abdominal flap donor by superficial epigastric arteriovenous, and the black area in the red circle means the thrombus. (e) - (f) Dorsal McFarlane flap vascular imaging and the black area in the red circle represented thrombus.


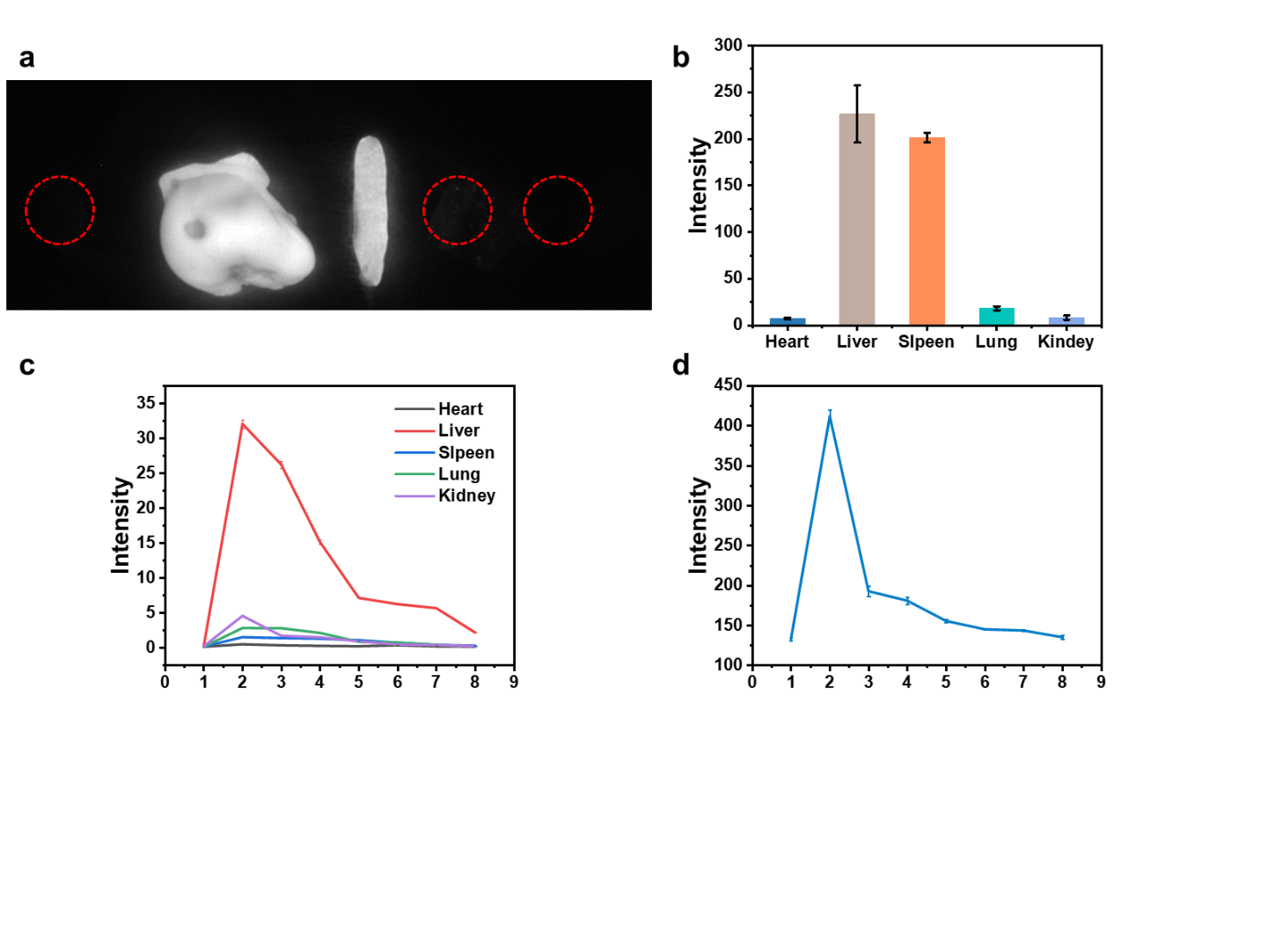


**Figure S6.** (a) Distribution of nanoparticles in organs. (b) Quantitative statistics in (a). (c) Organ metabolism. (d) Blood metabolism. Group 1 in (c) and (d) represents the blank group, 2-8 represent 2h, 4h, 8h, 16h, 1d, 3d, and 5d after injection.
